# Can we trust untargeted metabolomics? Results of the metabo-ring initiative, a large-scale, multi-instrument inter-laboratory study

**DOI:** 10.1007/s11306-014-0740-0

**Published:** 2014-10-14

**Authors:** Jean-Charles Martin, Matthieu Maillot, Gérard Mazerolles, Alexandre Verdu, Bernard Lyan, Carole Migné, Catherine Defoort, Cecile Canlet, Christophe Junot, Claude Guillou, Claudine Manach, Daniel Jabob, Delphine Jouan-Rimbaud Bouveresse, Estelle Paris, Estelle Pujos-Guillot, Fabien Jourdan, Franck Giacomoni, Frédérique Courant, Gaëlle Favé, Gwenaëlle Le Gall, Hubert Chassaigne, Jean-Claude Tabet, Jean-Francois Martin, Jean-Philippe Antignac, Laetitia Shintu, Marianne Defernez, Mark Philo, Marie-Cécile Alexandre-Gouaubau, Marie-Josephe Amiot-Carlin, Mathilde Bossis, Mohamed N. Triba, Natali Stojilkovic, Nathalie Banzet, Roland Molinié, Romain Bott, Sophie Goulitquer, Stefano Caldarelli, Douglas N. Rutledge

**Affiliations:** 1 INRA UMR1260, “Nutrition, Obésité et Risque Thrombotique”, 13385 Marseille, France; 2 Faculté de Médecine, Aix-Marseille Université, 13385 Marseille, France; 3 INSERM, UMR1062 “Nutrition, Obésité et Risque Thrombotique”, 13385 Marseille, France; 4 INRA, UMR 1083 SPO, INRA Campus SupAgro, Plateforme Polyphénols, 2 Place Viala, 34060 Montpellier Cedex 02, France; 5 BRUKER, 4 allée Hendrick Lorentz, 77447 Marne La Vallée Cedex 2, France; 6 INRA, UMR 1019, UNH, CRNH Auvergne, 63000 Clermond-Ferrand, France; 7 INRA, UMR 1019, Plateforme d’Exploration du Métabolisme, UNH, 63000 Clermond-Ferrand, France; 8 INRA, UMR 1331 TOXALIM (Research Center in Food Toxicology), Axiom-Metatoul, 31027 Toulouse, France; 9 Laboratoire d’Etude du Métabolisme des Médicaments, DSV/iBiTec-S/SPI, CEA-Saclay, 91191 Gif-sur-Yvette Cedex, France; 10 European Commission, Joint Research Centre, Institute for Health and Consumer Protection, Via Enrico Fermi 2749, 21027 Ispra, Italy; 11 INRA, UMR1332 Fruit Biology and Pathology, Centre INRA de Bordeaux, 33140 Villenave d’Ornon, France; 12 INRA, UMR 1145 Ingénierie Procédés Aliments, 75005 Paris, France; 13 AgroParisTech, UMR 1145 Ingénierie Procédés Aliments, 75005 Paris, France; 14 UPMC, Institut Parisien de Chimie Moléculaire, UMR-CNRS 7201, 4 Place Jussieu, Paris Cédex 05, France; 15 INRA, UMR 1331 TOXALIM (Research Center in Food Toxicology), Metabolism of Xenobiotics (MeX), 31027 Toulouse, France; 16 LUNAM Université, Oniris, Laboratoire d’Etude des Résidus et Contaminants dans les Aliments (LABERCA), USC INRA 1329, BP 50707, 44307 Nantes Cedex 3, France; 17 Institute of Food Research, Norwich Research Park, Norwich, NR4 7UA UK; 18 Aix-Marseille Université, ISM2, Campus Scientifique Saint Jérôme, 13397 Marseille Cedex 20, France; 19 Université Paris 13, Sorbonne Paris Cité, Laboratoire CSPBAT, CNRS (UMR 7244), 93017 Bobigny, France; 20 LCH, Laboratoire des Courses Hippiques, 91370 Verrières-le-Buisson, France; 21 AP-HM, Hôpital Timone, Laboratoire de Biochimie, 13385 Marseille, France; 22 Université de Picardie Jules Verne, EA 3900 BIOPI Biologie des plantes innovation, UFR de Pharmacie, 1 rue des Louvels, 80000 Amiens, France; 23 MetaboMer, FR2424, CNRS/UPMC, Station Biologique de Roscoff, Place Georges Tessier, 29680 Roscoff, France

**Keywords:** Inter-laboratory, Untargeted metabolomics, Mass spectrometry, Nuclear magnetic resonance, Metabolic fingerprinting

## Abstract

The metabo-ring initiative brought together five nuclear magnetic resonance instruments (NMR) and 11 different mass spectrometers with the objective of assessing the reliability of untargeted metabolomics approaches in obtaining comparable metabolomics profiles. This was estimated by measuring the proportion of common spectral information extracted from the different LCMS and NMR platforms. Biological samples obtained from 2 different conditions were analysed by the partners using their own in-house protocols. Test #1 examined urine samples from adult volunteers either spiked or not spiked with 32 metabolite standards. Test #2 involved a low biological contrast situation comparing the plasma of rats fed a diet either supplemented or not with vitamin D. The spectral information from each instrument was assembled into separate statistical blocks. Correlations between blocks (e.g., instruments) were examined (RV coefficients) along with the structure of the common spectral information (common components and specific weights analysis). In addition, in Test #1, an outlier individual was blindly introduced, and its identification by the various platforms was evaluated. Despite large differences in the number of spectral features produced after post-processing and the heterogeneity of the analytical conditions and the data treatment, the spectral information both within (NMR and LCMS) and across methods (NMR vs. LCMS) was highly convergent (from 64 to 91 % on average). No effect of the LCMS instrumentation (TOF, QTOF, LTQ-Orbitrap) was noted. The outlier individual was best detected and characterised by LCMS instruments. In conclusion, untargeted metabolomics analyses report consistent information within and across instruments of various technologies, even without prior standardisation.

**Electronic supplementary material:**

The online version of this article (doi:10.1007/s11306-014-0740-0) contains supplementary material, which is available to authorized users.

## Introduction

Metabolomics has become essential to understanding the impact of external or pathological stressors on a biological system (Ryan and Robards 2006). Although early attempts at using such analytical approaches took place in the 1970s (Pauling et al. 1971), metabolomics approaches have increased only since the beginning of this century and have appeared promising only during the present decade (Opinion 2010). For instance, recent studies have transitioned metabolomics from proof-of-principle to validation. In these studies, untargeted metabolomics allowed a hypothesis to be generated and to be challenged in order to validate new biomarkers of disease (Wang et al. 2011; Cobb et al. 2013), which ultimately led to the development of a clinical test (Cobb et al. 2013). However, the implementation of metabolomics is not trivial and requires validation and an estimation of reliability, even though some standardisation attempts have been made and recommendations have been proposed (Fiehn et al. 2006; Lindon et al. 2005; Scalbert et al. 2009). This is an important issue because, in addition to sampling (Griffin et al. 2007) and extraction procedures (Want et al. 2006; Tulipani et al. 2012; Pereira et al. 2010), the performance of metabolomics analyses also relies on the instrument type (Gika et al. 2010; Williams et al. 2005; Rubtsov et al. 2007) and on the methods implemented (van den Berg et al. 2006; Tautenhahn et al. 2008; Sumner et al. 2007). Such heterogeneity can thus result in discrepancies in the results produced from different places and prevent their generalisation. Several inter-laboratory studies have attempted to validate the accuracy of the metabolomics approach, but these studies all used similar instruments, either NMR of different magnetic fields (Viant et al. 2009; Ward et al. 2010) or GC–MS (Allwood et al. 2009) and LC–MS (Benton et al. 2012) of the same type. When using tight standardized conditions among the partners these studies showed a high degree of inter-laboratory repeatability. However they did not address comparisons of heterogeneous instruments or methods nor the fact that strict protocol designs are difficult to extrapolate to real-life situations. Comparisons among various instruments can also be challenging because metabolite coverage is highly instrument-dependent (Mandal et al. 2012; Suhre et al. 2010). The best starting point and limiting point in the metabolomics analytical workflow is to compare the spectral information gathered from instruments of various technologies irrespective of the samples preparation step. This issue was recently addressed at the intra-laboratory scale using two types of LCMS systems of different technologies (Gika et al. 2010; Glauser et al. 2013). The authors found good convergence between the instruments, but the results of these intra-laboratory studies needs to be challenged at the inter-laboratory scale and to be extended to heterogenous instruments to allow complete generalisation.

Thus, to respond to this challenge and to facilitate standardisation initiatives, it would be wise to determine the usefulness of the current metabolomics strategies in delivering homogeneous results using both homologous and heterologous instrumentation and methods. To this end, we designed 2 metabolomics ring-tests (Test #1 and Test #2), in which the same sets of samples were analysed without any imposed standardisation on 16 instruments (5 NMR and 11 LCMS) located in three European countries. These two tests included a spiking experiment in human urines and plasma analysis of rats challenged with vitamin D. Our primary goal was not to make comparisons at the analytes level but rather to evaluate the inter-instrument convergence at the metabolic profiling level. A specific statistical design was applied to make these comparisons possible. Both instruments (NMR and LCMS) and procedures used covered the most common situations observed in non-targeted metabolomics.

## Materials and methods

### Experimental setup

#### Test #1: High biological contrast

Fourteen volunteers were recruited, including 13 males (age 17–50) and one woman in mid-pregnancy (age 32). The study was approved by the regional committee on human experimentation (No. 2008-A01354–51, Comité de Protection des Personnes Sud Méditerranée I). A written informed consent for the use of the urine samples was signed by each individual. The sample list was sent blind to all analytical partners.

The specific gravities of the urine samples were determined using a density meter (Anton Paar, Austria) to calculate a normalisation factor to be applied to each signal intensity measured by NMR or LCMS (Cone et al. 2009) whenever specified.

A mixture of standards was prepared to be incorporated into the urine samples using the NuGO standard operating procedure (SOP) number 43 produced by the University of Copenhagen, details of which are available via the web link: http://www.nugo.org/frames.asp?actionID=39148&action=loginFromPP. For further information, please contact Lars Dragsted ldra@life.ku.dk. The standard mixture only contained 32 chemicals (see online resource Table 1): ascorbic acid, citrulline, creatinine, taurine, uric acid, caffeine, glutaric acid, inosine, isoleucine, leucine, pyroglutamic acid, methionine, methylmalonic acid, *N*-methylhistidine, aminobenzoic acid, phenylalanine, proline, riboflavin, adenosine, adenine, adipic acid, azelaic acid, caffeic acid, tryptophan, tyrosine, uracil, uridine, chenodeoxycholic acid, cholic acid, cortisone, deoxycholic acid, glycocholic acid. All the molecules can be naturally present in urine, except chenodeoxycholic acid.

To prevent dilution the standard mixture was aliquoted then lyophilised and reconstituted with each urine sample.

#### Test #2: low biological contrast

Vitamin D is involved in many biological functions and in the maintenance of health (Adams and Hewison 2010). It occurs endogenously but can also be provided by various food sources. We chose to examine its metabolic effect as a test of a low biological contrast study, which is a common situation in nutritional metabolomics research. Twenty Sprague–Dawley male rats, weighing 250 g and purchased from JANVIER SAS (Le Genest Saint Ile, France), were fed for 6 weeks after 1 week of acclimatisation while maintained in a dark/light cycle of 12 h. All experiments were conducted according to the French Regulations for Animal Experimentation (Art 19. Oct 1987, Ministry of Agriculture) and in conformity with the Public Health Service Policy after approval by our institutional Animal Care and Use Committee. Half of the rats were fed with the low vitamin D diet (1,000 IU/kg of diet, SIGMA ref C9756, L’Isle d’Abeau Chesnes, France), while the others received a high vitamin D diet (20,000 IU/kg of diet) (online resource Table 2), as described elsewhere (Fleet et al. 2008). After 6 weeks, the rats were anesthetised with isoflurane and exsanguinated through the left ventricle with a heparinised syringe. The blood was immediately cooled to 0 °C, and the plasma separated at 3,000 g and 4 °C for 10 min. For LCMS analysis, plasma deproteinisation and metabolite extraction were performed by methanol treatment, as described by (Pereira et al. 2010); 500 µL plasma samples were kept unprocessed for NMR analysis. All the operations were performed on randomly ordered samples. We checked that the supplementation increased the vitamin D in the plasma by assaying the 25(OH)-vitamin D3 concentration. As a result of the supplementation, the rats weighed more after 6 weeks (552 ± 9 g versus 517 ± 5 g in the supplemented versus deprived rats, respectively; *P* < 0.05) and the 25(OH)-vitamin D3 concentration was significantly increased (215 ± 8 and 63 ± 8 mmol/L in supplemented vs deprived rats, respectively). Extracted (for LCMS) or unprocessed (for NMR) plasma samples were aliquoted into Eppendorf tubes and shipped to participants in dry ice along with QC samples and blank samples. Once received, the samples were stored at −80 °C until analysis within 1–3 months.

### Metabolomics analysis

The instruments used in both tests were, for NMR, a Bruker 500 Avance III, a Bruker DRX-600 Avance, and three Bruker Avance III 600, and for LCMS (all operating in ESI), a Bruker microTOFQ coupled to an Agilent RRLC, a Bruker microTOFQII coupled to an Ultimate 3000 Dionex U-LC, a Bruker QTOF Maxis Impact coupled to an Ultimate 3000 Dionex U-LC, a Waters QTOF Premier coupled to an Acquity UPLC, a Waters QTOF Micro coupled to an Acquity UPLC, a Thermo Fisher Scientific LTQ-Orbitrap coupled to an Agilent 1200 RRLC, a Thermo Fisher Scientific LTQ-Orbitrap Discovery coupled to an Ultimate 3000 Dionex U-LC, a Thermo Fisher Scientific LTQ-Orbitrap Discovery coupled to an Accela liquid chromatographic system (Thermo Fisher Scientific, Les Ulis, France), a Thermo Fisher Exactive coupled to a Shimadzu Nexera liquid chromatography system, a Waters QTOF Synapt-2 MS coupled to a ThermoAccela binary UPLC, and a Bruker Micro-TOF delivered by an Agilent 1100 LC.

For both NMR and LCMS analyses, each participating laboratory was asked to use its own in-house protocols for instrument tuning, data processing and post-processing. A detailed description of all the procedures used by the different platforms is given in the online resource. Analyses were performed in random order (LCMS and NMR). The same quality control sample, consisting of a pool of urine (Test #1) or plasma (Test #2) samples, was provided to each partner and was analysed by insertion into the analytical series (from every 5 to every 10 samples) to check the performance of the analytical system in terms of retention times, accurate mass measurements, and signal intensities (all LCMS). The analytical variability compared to the biological variability was assessed using these quality controls samples. The low dispersion (almost nill with NMR) of the QC samples obtained by each partner after PC analysis indicated proper analytical conditions.

### Statistical analysis

The statistical analyses were applied separately for Test #1 and Test #2. The Test #1 samples were analysed by 14 instruments (5 NMR and 9 LCMS operating in positive and negative modes), whereas in Test #2, 12 instruments (4 NMR and 8 LCMS operating mainly in positive mode) were used (Table 1).

**Table 1:** Descriptions of the MS and NMR platforms used in the two tests and the number of features retained per test and instrument

Platform ID^1^	Instruments	Mode	Deconvolution software	Test #1 *n* = 25	Test #2 *n* = 18	Stat
N1	Bruker 600	–	AMIX	751	881	Multivariate
N2	Bruker 600	–	AMIX	252	–	Univariate and multivariate
N3	Bruker 600	–	AMIX	88	9300	Multivariate
N4	Bruker 500	–	In-house	9,699	9550	Multivariate
N5^a^	Bruker 600	–	AMIX	233	120	Multivariate
O1P	LTQ orbitrap	Positive	XCMS	5,035	710	
Q6P/O2P^b^	QTOF premier/orbitrap	Positive	XCMS	1,922	1295	Univariate
Q6N	QTOF premier	Negative	XCMS	314	–	Univariate
O3P	LTQ orbitrap	Positive	XCMS	1,827	1979	Univariate
O3N	LTQ orbitrap	Negative	XCMS	1,715	795	Univariate
O4P	LTQ orbitrap	Positive	XCMS	2,668	–	Multivariate
Q1P	QTOF micro	Positive	XCMS	1,181	504	Multivariate
Q1N	QTOF micro	Negative	XCMS	1,288	–	Multivariate
Q2P	QTOF impact	Positive	XCMS	1,688	–	Multivariate
Q2N	QTOF impact	Negative	XCMS	2,492	–	Multivariate
Q3P	microQTOF	Positive	XCMS	908	2631	Univariate
Q4P	QTOF micro II	Positive	XCMS	909	2277	Multivariate
Q4N	QTOF micro II	Negative	XCMS	438	–	Multivariate
Q5P^a^	QTOF synapse	Positive	XCMS	6,992	1595	Univariate
Q5N^a^	QTOF synapse	Negative	XCMS	5,167	–	Univariate
T1P^a^	TOF	Positive	XCMS	580	–	Univariate
T1N^a^	TOF	Negative	XCMS	398	–	Univariate

^a^These instruments are located on the same platform

^b^This platform used a QTOF analysis in Test #1 and an Orbitrap analysis in Test #2

^1^N for NMR spectrometer, Q for QTOF mass spectrometer, O for orbitrap mass spectrometer, T for TOF mass spectrometer. The P or N appended to the mass spectrometer identifier number denotes positive or negative ionisation mode, respectively

A workflow of the statistical design is presented in the online resource Figure 1.

For each test data from all the platforms were pooled, and one statistical analysis each was applied. The aim of this global statistical analysis was to assess the convergence of the biological information delivered by the metabolic profiling and provided by several instruments (NMR and LC–MS). All statistical methods used to extract the common information shared by all the platforms are detailed in the online resource. Briefly, we compared the relationship between the data tables using RV coefficients (Escoufier 1973; Lavit et al. 1994), which can be interpreted as the multivariate equivalent of a squared correlation coefficient (*R* ^2^) ranging from [0–1]. An RV coefficient equal to 1, when considering the two tables X and Y, means that the relative position of the samples in X is similar to those in Y. In other words, the information included in the two data tables is identical. We also explored the common information among the various data tables using the Common components and specific weights analysis (i.e., CCSWA or ComDim). This method was developed by Qannari et al. in 2000 (Qannari et al. 2000) for sensory profiling analysis and has also been applied to chemometrics studies (Mazerolles et al. 2002). Several extensions of the method have been developed recently (Amat et al. 2010; Jouan-Rimbaud Bouveresse et al. 2011; Mazerolles et al. 2006). CCSWA estimates the dispersion of the samples in a series of dimensions that are common to all the data tables. Each data Table has a specific weight (called salience) that quantifies its contribution to each common dimension. Based on global scores, samples can be projected onto the common space to determine the sample structures that are common to all data tables. The CCSWA algorithm has been described elsewhere (Bro et al. 2008; Qannari et al. 2000).

The RV coefficient matrices and CCSWA were computed using the SAISIR package developed for the open source SCILAB software (Bertrand and Cordella 2008).

Our secondary endpoint was to identify a set of features allowing discrimination of the two groups in each test. For Test #1, the additional objective for all partners was to identify all of the 32 molecules added to the spiked samples. Table 1 summarises the statistical methods used by each laboratory for identifying the discriminating features in both tests. The univariate statistics refer to the significance of the fold-change, whereas the multivariate methodology refers to the PLS-DA regression. The discriminating features found by each partner were also compared to the ones found in the CCSWA performed on the post-processing datasets computed in the data collection centre.

#### Correlation networks

The correlation network is an efficient tool for providing a graphical representation of the correlations between variables. In this study, the correlation network visualises multiple proximities between instruments based on the estimated RV coefficients. The RV coefficient networks were calculated and visualised using the Cytoscape software (Shannon et al. 2003) (http://www.cytoscape.org/).

### Characterisation of the Test #1 outlier

The CCSWA performed in the data collection centre was applied to select spectral features present in the NMR and LCMS analyses that discriminated the blind biological outlier individual introduced among the urine samples. For annotation purposes, the features retained were selected according to a correlation value, with the common component characterising the outlier as being more than 0.8 for LCMS or more than two z-scores for NMR. This biological outlier was a mid-term pregnant woman contrasting with the samples collected from male individuals.

### Annotation of discriminating features

Annotation was either performed after operator visual spectral inspection or using automated procedures based on accurate mass and referencing to public or in-house databases (MS instruments). Metabolites identification referred to level 1 of the MSI when dealing with the spiked standards, or to level 2 whenever applicable, and thus corresponded to putative annotation (Sumner et al. 2007).

## Results

### Sample analysis, data acquisition and post-processing

The procedures used for data acquisition and data post-processing and filtering prior to the statistical analyses are detailed in the online resource.

For NMR, the operators used AMIX or in-house C programs to bin the spectra into regions of various ppm widths (from 0.001 to 0.04 ppm).

For LCMS, all partners used XCMS for peak picking and retention time correction; however, the basal parameters and methods scripts differed among the partners based on their individual background experience. This led to different workflows for XCMS from one platform to the other. In addition, further signal filtering was sometimes applied, such as de-isotoping and de-adducting, de-noising using blank samples or QC dilutions, elimination of unreliable features based on the use of QC samples, etc. (see online resource).

As a result, the various non-standardised, in-house procedures applied to the heterogeneous analytical platforms led to very different datasets in terms of the number of features retained and the characteristic features for the two tests (Table 1). For instance, the number of features varied by 2 orders of magnitude from the lowest to the highest dimensional data Table (from 88 to 9699 for Test #1 and from 120 to 9550 for Test #2). The number of features was less heterogeneous within the LCMS instruments (from 398 to 6992 in Test #1 and from 504 to 2271 in Test #2).

### Group discrimination by statistical analyses

**Fig. 1:**
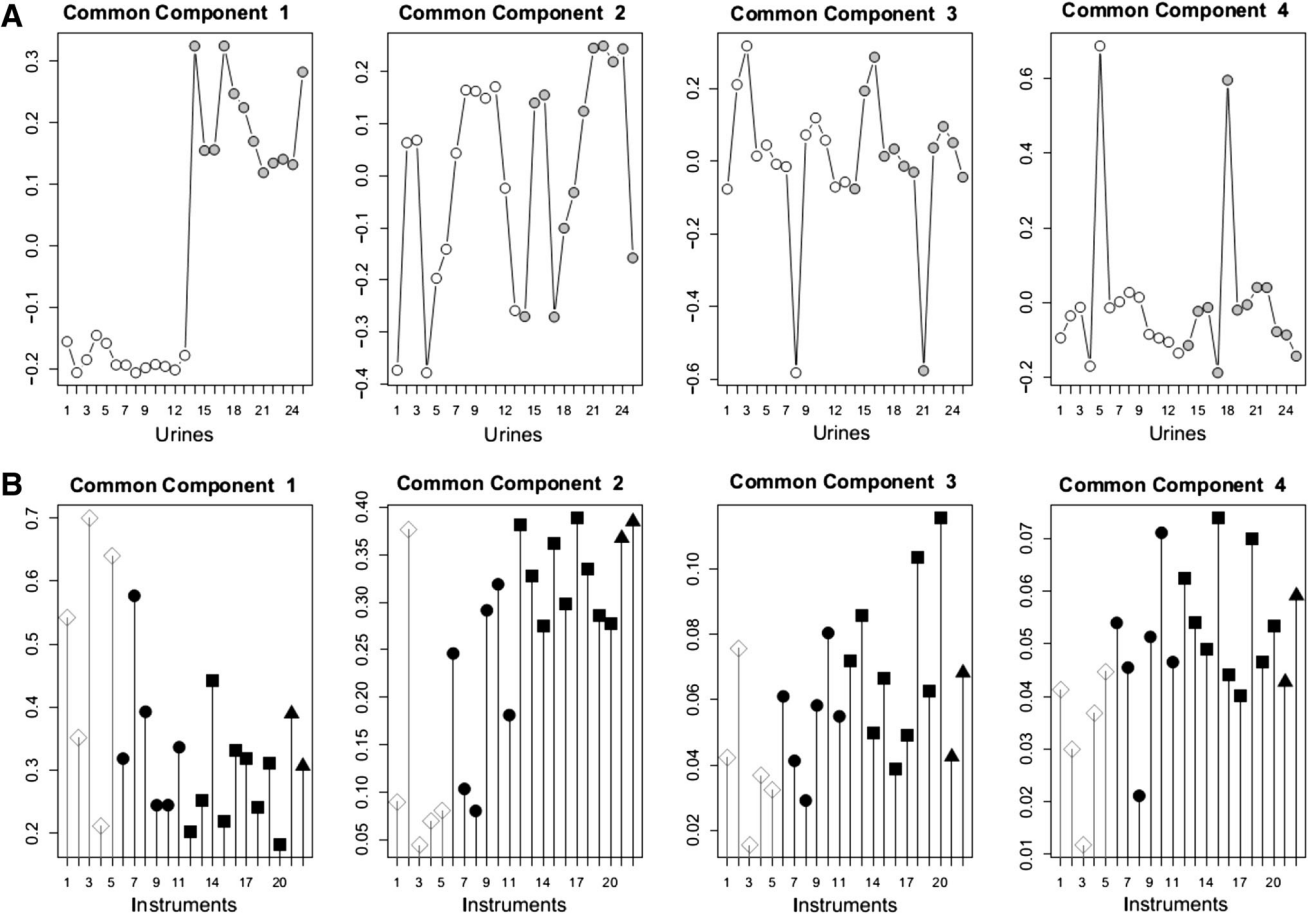
Individual common component and specific weights analysis scores (**a**) and saliences or loadings (**b**) calculated from the Test #1 datasets. The first common component discriminates the control group from the spiked group. The second common component describes the inter-individual variability that was most common to all the instruments. The third and fourth components highlight the specific metabolic profiles of urine samples 8 and 5, respectively. For the NMR instruments (NMR1, NMR3 and NMR5), the major part of the variability (i.e., 70 %) explained the discrimination between the two groups. NMR instruments characterised 10 % of the total variability as inter-individual variability (saliences on the second common component). Among the LCMS instruments, 20 to 50 % of the total variability explained the inter-group variability (first component), and 20 to 30 % of the total variability explained the inter-individual variability. NMR2 was different from the other NMR instruments because it was associated with, respectively, 35 and 38 % of the total inter-group and inter-individual variability. Two particular urine samples (individuals #5 and #8) were identified by all LCMS instruments, and only NMR2 identified individual #8, in the same proportion (approximately 5 % of total variability). **a** *Open circles* denote the original urine samples, *grey circles* denote the matched spiked samples. **b** *Open diamond* denotes NMR, *black circles* denote Orbitrap,* squares* denote QTOF, and* triangles* denote TOF

In Test #1 (high contrast), all the platforms used their own statistical analyses and all partners were able to discriminate the spiked group from the non-spiked group (Tables 1, 2). The CCSWA performed by the referent partner brought further external validation of each partner’s findings (Table 2). Using either ESI–MS approaches and the exact mass of the monoisotopic ion and isotopic distribution or one-dimensional ppm shifts (NMR), from 16 to 23 were identified by NMR, and from 13 to 25 of the 32 spiked standards were identified by LCMS (both polarity modes) (see online resource Table 3). From 36 to 133 discriminating features for NMR and from 74 to 2215 features for LCMS were thus retained by the partners and characterised the spiked molecules. The proportions of discriminating features produced varied from 11 to 57 % in the NMR instruments and from 5 to 33 % in the LCMS instruments (from Table 2 data). The CCSWA calculated from the combination of all the data tables confirmed the partner’s statistical analyses, with a clear discrimination between the spiked and non-spiked urine samples found by all platforms (Fig. 1a and online resource Figure 5A).

**Table 2:** Results of Test #1 for the various platforms

Platform ID^1^	Mode	Total features	Discriminating features from independent analysis	Number of identified standard molecules^2^	Discriminating features from CCSWA (*r* > 0.8)	Features shared by both methods	%^1^ of shared features	Discriminating features from CCSWA (*r* > 0.9)	Features shared by both methods	%^3^ of shared features
N1	–	751	86	22	188	65	35	103	50	49
N2	–	252	93	24	43	33	77	30	22	73
N3	–	88	36	16	37	23	62	21	13	62
N4	–	9,699		NR	345	–		66		
N5^a^	–	233	133	23	110	68	62	60	43	72
O1P	Pos	5,035		25	798	–		552		
Q6P	Pos	1,922	354	22	820	102	12	398	38	9.5
Q6N	Neg	314	137	14	69	43	62	27	19	70
O3P	Pos	1,827	265	23	194	194	100	158	158	100
O3N	Neg	1,715	348	25	222	222	100	177	177	100
O4P	Pos	2,668		11	433	–		256		
Q1P	Pos	1,181	118	21	129	108	84	104	95	91
Q1N	Neg	1,288	210	13	171	171	100	152	152	100
Q2P	Pos	1,688	440	NR	472	415	88	388	373	96
Q2N	Neg	2,492	153	NR	268	149	56	203	143	70
Q3P	Pos	908	167	13	184	83	45	144	66	46
Q4P	Pos	909	202	23	176	85	48	151	74	49
Q4N	Neg	438	74	15	58	58	100	52	52	100
Q5P^a^	Pos	6,992	2215	20	1431	1430	100	970	970	100
Q5N^a^	Neg	5,167	1137	22	475	474	100	257	257	100
T1P^a^	Pos	580	192	22	139	139	100	117	117	100
T1N^a^	Neg	398	101	14	77	77	100	66	66	100

Total number of features, number of discriminating features per statistical method and number of discriminating features shared by the two statistical methods for each instrument

^a^These instruments are located on the same platform

^1^N for NMR spectrometer, Q for QTOF mass spectrometer, O for Orbitrap mass spectrometer, T for TOF mass spectrometer. The P or N appended to the mass spectrometer identifier number denotes positive or negative ionisation mode, respectively

^2^Annotated standard molecules detected by each laboratory are reported in online resource Table 3; annotation was performed only on features that statistically differed in the spiked samples, except for partners O1, Q2 and N4 (*NR* for non-reported)

^3^The percentage of discriminating features (selected by the CCSWA) that were also selected as discriminating by the simple statistical analysis carried out by each participating laboratory

However, the NMR platforms generally performed better than LCMS in distinguishing the spiked versus non-spiked urine, with an average percentage variance (CC1 score) of 0.494 ± 0.092 compared to 0.316 ± 0.026 for LCMS (*P* = 0.0159) (Fig. 1b). Conversely, the LCMS platforms showed higher CC scores than did NMR in depicting interindividual metabotypes (0.285 ± 0.092 and 0.132 ± 0.061 in CC2 for LCMS and NMR, respectively, *P* = 0.0093, Fig. 1b). As a result, all the LCMS instruments were equally proficient at distinguishing interindividual and intergroup variability, whereas NMR mainly described intergroup variability, except NMR2, which shared closer characteristics with the LCMS platforms than with the other NMR instruments. Of note is that the relative score distribution pattern summarizing the individual metabotypes was not modified in the spiked versus native urine samples in Test #1 either for NMR or LCMS (Figs. 1a and 5A).

The urine of a mid-term pregnant woman introduced as a blind outlier (individual #5) was detected in the common space component 4 (Fig. 1a), but this occurred mainly with LCMS (variance described in CC4 was 0.051 ± 0.003 for LCMS and 0.033 ± 0.006 for NMR, *P* = 0.0113) (Fig. 1b). Based on the exact mass only or on the exact mass and isotopic distribution of the discriminating features, most of the LCMS instruments found that the difference between this individual and the others was due to estro-progestative components in the urine (online resource Table 4). On the other hand, NMR found that the discrimination was due to alanine, lactate, glycine, glutamine, and threonine, among the most consensual metabolites (online resource Table 4), as determined from chemical shifts and database matching. Of note is that another outlier (individual # 8) was unexpectedly revealed in CC3 of Fig. 1a. It was also detected by most of the LCMS instruments and by NMR2 (Fig. 1b).

**Fig. 2:**
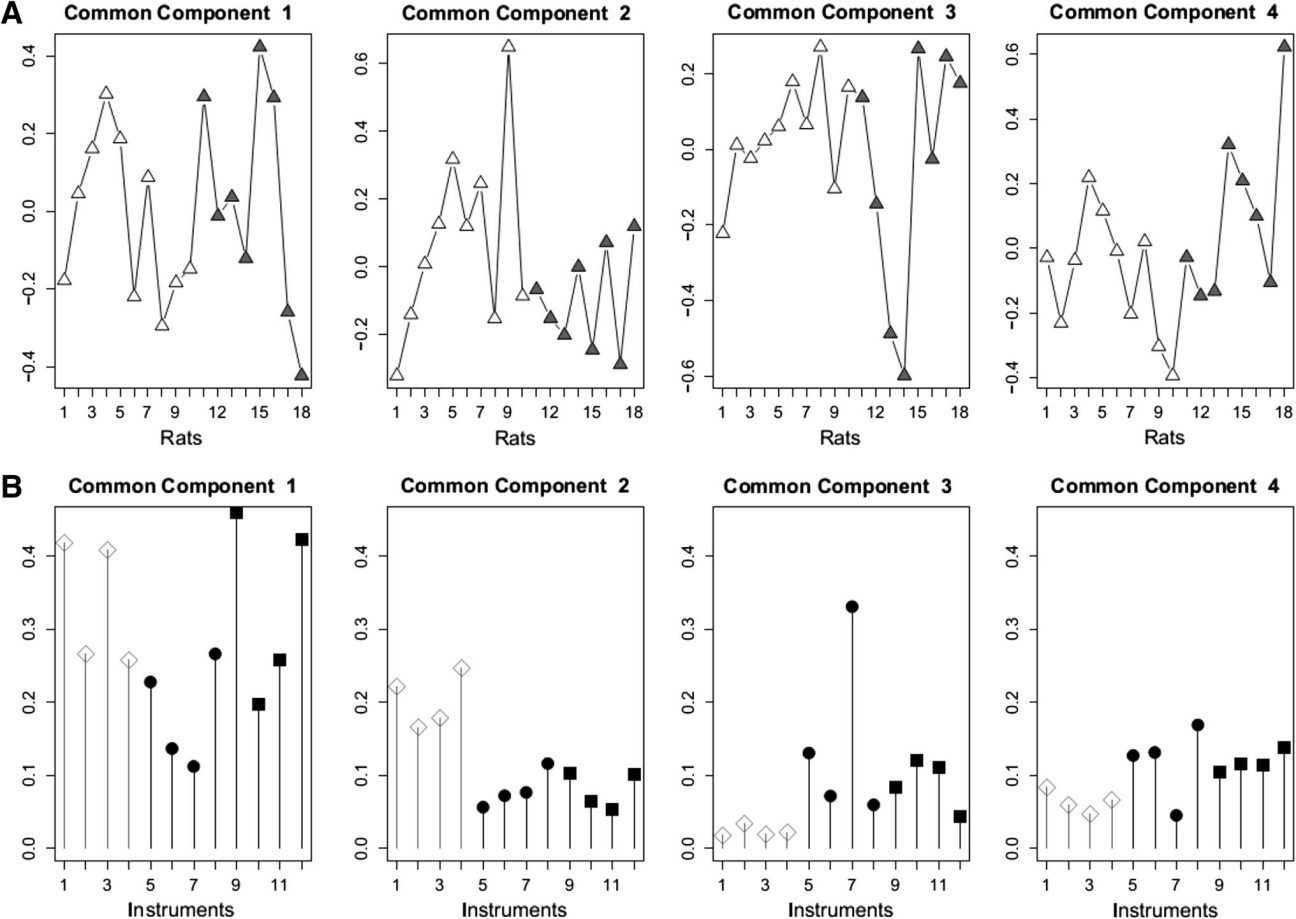
Individual common component and specific weights analysis scores (**a**) and saliences or loadings (**b**) calculated from the Test #2 datasets. No common dimension discriminated the groups, although common component #2 showed a trend towards group discrimination. More than 50 % of the total variability of the NMR instruments and of 2 LCMS instruments (Q2P and Q6P) was recovered in the first two common dimensions. The variability of all other LCMS instruments was mainly in the third and the fourth common components. The structure of the samples associated with the second common component is essentially due to the NMR instruments

In Test #2 (low biological contrast), no discrimination between the groups was determined based on the signals detected by each instrument unless specific signal correction was applied (orthogonalisation to discard part of the variance that was not linked to class characteristics) (Fearn 2000; Trygg and Vold 2002). The vitamin D specific biological effect was thus not measured as the major part of the biological variance. Even if the two groups were not statistically distinguishable, it was interesting to compute the overall statistical analysis in order to estimate the common biological information extracted by several instruments and revealing individual metabotypes. The CCSW analyses also confirmed the partner’s statistical findings showing at best a tendency to distinguish among the supplemented versus non supplemented vitamin D rats (Fig. 2a and online resource Figure 5B).

For both tests, looking at online resource Figure 5, each sample was represented in the common space (plot) as the barycentre of all the individual platform results, with the lines beaming towards the scores of the individual platforms showing their dispersion around the barycentre. In Test #1, this representation indicated that LCMS presented less dispersion than NMR in depicting individual metabotypes (score 2, online resource Figure 5A), whereas NMR showed less dispersion in extracting intergroup differences (score 1). This was no longer observed for the low contrast plasma samples of Test #2 (online resource Figure 5B).

#### Estimation of the statistical link between instruments

The RV coefficient matrix was calculated on 22 data sets for Test #1 and on 12 data sets for Test #2. For both tests, the estimated average links (i.e., RV coefficient) of each instrument with all of the others are shown in online resource Fig. 2. RV coefficients can show artificially high values when comparing megadata sets (Smilde et al. 2009). We thus compared the observed to the re-sampled RV values (Online resource Figure 2) and to the modified RV-coefficient for large datasets designed by Smilde et al. (2009) (online resource Figure 2). The observed RV values were generally much higher than the random ones generated in both tests (online resource Figure 2) and were close to the modified-RV (online resource Fig. 2), especially in Test #1.

The RV values can define the convergence between the instruments in our study, and is thus the ability of each instrument not to deviate in reporting the individual metabolomes in the multivariate space.

**Fig. 3:**
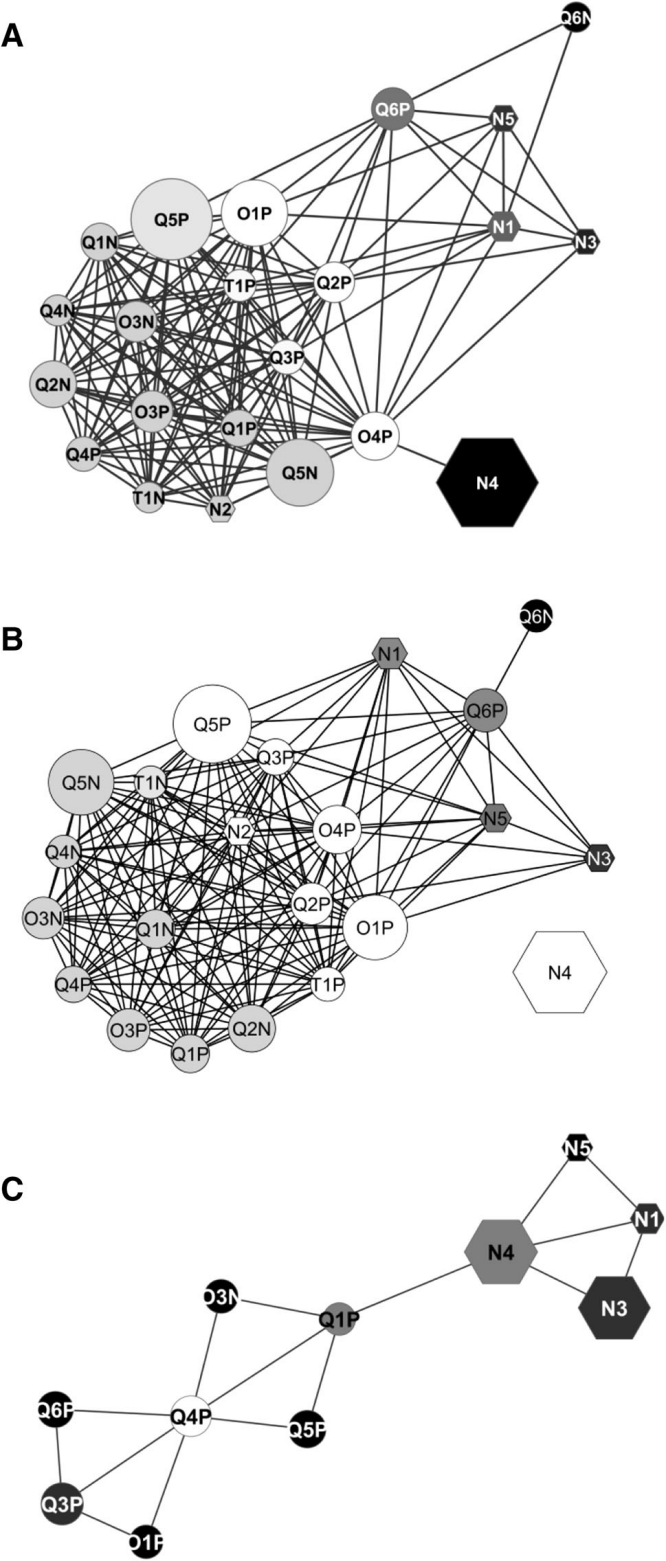
Correlations networks calculated from the pair-wise RV coefficients matrix from Test #1 (**a**) with spiked and non-spiked samples or with native urine samples only (**b**) and from Test #2 (**c**). Node labelling: *N* NMR platforms, *Q* QTOF mass spectrometer, *O* orbitrap mass spectrometer, *T* TOF mass spectrometer. The P or N appended to the mass spectrometer identifier number denotes positive or negative ionisation mode, respectively. *Node shapes*: hexagon for nuclear magnetic resonance platforms, ellipse for mass spectrometers. The node size is proportional to the number of features retained by each instrument. The node colour from *black* to *white* indicates an increasing node degree (number of edges per node). The edges represent the RV coefficient values, with cut off values ≥0.791 in Test #1 and ≥0.708 in Test #2). At this cut off level, O3P was excluded from the Test #2 network (**b**)

In Test #1, the convergence of the metabolic profiles between instruments was much better than in Test #2. Thus, the average RV coefficients in Test #1 were close to 0.8 (online resource Figure 2) for all instruments, except for NMR4 (0.67) and the Orbitrap LCMS O2N operating in negative mode (0.64). The NMR2 instrument was more similar to all the LCMS instruments (average RV equal to 0.90) than to the other NMR instruments (average RV equal to 0.68). To discard the artificial convergence that may arise from the addition of the standard metabolites, the RV were recalculated while excluding the spiked urine samples. The closeness between the instruments based on the RV values was not modified, as shown in Table 3 and in Fig. 3b.

**Table 3:** Average RV coefficients within the NMR and MS instruments and the average RV coefficients between the NMR and MS instruments for Test #1 and Test #2

Instrument^2^	Within-methods^1^ RVs			Between-methods RVs		
	Test #1 (*n* = 25)	Test #1^3^ (*n* = 25)	Test #2 (*n* = 18)	Test #1 (*n* = 25)	Test #1^3^ (*n* = 25)	Test #2 (*n* = 18)
NMR						
N1	0.86	0.85	0.84	0.74	0.80	0.56
N2	0.68	0.74	–	0.88	0.90	–
N3	0.82	0.79	0.72	0.68	0.70	0.51
N4	0.71	0.63	0.80	0.67	0.60	0.57
N5*	0.85	0.85	0.78	0.72	0.79	0.52
Average	0.78	0.77	0.78	0.74	0.76	0.54
MS						
O1P	0.91	0.92	0.68	0.80	0.84	0.56
Q6P/O2P^a^	0.76	0.76	0.64	0.88	0.86	0.52
Q6N	0.64	0.66	–	0.73	0.72	–
O3P	0.89	0.90	0.44	0.71	0.74	0.31
O3N	0.91	0.91	0.60	0.70	0.73	0.46
O4P	0.88	0.89	–	0.85	0.85	–
Q1P	0.87	0.87	0.70	0.61	0.65	0.65
Q1N	0.90	0.90	–	0.69	0.71	–
Q2P	0.90	0.90	–	0.85	0.85	–
Q2N	0.88	0.88	–	0.63	0.66	–
Q3P	0.92	0.92	0.67	0.79	0.81	0.55
Q4P	0.91	0.91	0.75	0.72	0.73	0.58
Q4N	0.89	0.91	–	0.67	0.70	–
Q5P*	0.92	0.89	0.66	0.78	0.79	0.66
Q5N*	0.87	0.93	–	0.64	0.67	–
T1P*	0.91	0.87	–	0.79	0.80	–
T1N*	0.90	0.91	–	0.71	0.74	–
Average	0.87	0.87	0.64	0.74	0.75	0.54

* These instruments are located in the same platform

^a^This platform used an Orbitrap analysis in Test #1 and a QTOF analysis in Test #2

^1^“Methods” refers to NMR and MS technologies

^2^N for NMR spectrometer, Q for QTOF mass spectrometer, O for Orbitrap mass spectrometer, T for TOF mass spectrometer. The P or N appended to the mass spectrometer identifier number denotes positive or negative ionisation mode, respectively

^3^Calculation made by excluding the spiked samples and based only on parent samples

In Test #2, all instruments had similar levels of concordance (average RV = 0.6), except for the LCMS instrument O3P (Orbitrap 3 in positive mode), which had an RV coefficient close to 0.4. This instrument clearly provided profiling information that did not converge with the others. Indeed, the distribution of the RV coefficients based on random re-sampling was close (median = 0.3) to the original RV coefficient.

When examining the RV coefficients calculated within methods (e.g., either among NMR or among LCMS), NMR showed somewhat less convergence in the profiling than did LCMS in Test #1, irrespective of the artificial contrast due to spiking (0.78 and 0.77 in NMR versus 0.87 and 0.87 for LCMS in spiked *vs* non-spiked samples, respectively). While the same values for Test #1 were found in Test #2 for NMR, the convergence between LCMS was lower in Test #2 (0.78 for NMR vs. 0.64 for LCMS) (Table 3). When examining the between methods RV coefficients (e.g., metabolic profiling convergence between NMR and LCMS), the values decreased from ~0.75 in Test #1 (urine samples) to 0.54 in Test #2 (rat plasma samples) (Table 3). Of note is that in Test #1, while QTOF Q6 (both in positive and negative ionisation modes) was in higher correlation to NMR than to the other LCMS, the reverse held true for NMR2, which was in higher correlation to LCMS than to the other NMR (Table 3). The RV coefficients matrix (closeness) calculated between each instrument was further visualised as an interaction network calculated for each test (Fig. 3). Cut-off values of RV = 0.791 and 0.708 were chosen in Test #1 and Test #2, respectively, to produce networks including all nodes with the least number of edges.

In the Test #1 network, most of the LCMS platforms clustered together, except for QTOF Q6, which was the LCMS platform sharing less common information with the other platforms, irrespective of spiking. Conversely, NMR N2 tightly clustered with most of the LCMS platforms, away from the NMR region of the network (Fig. 3a, b). Noticeably, when data normalisation was performed on the total area, as with the other NMR, instead of on trimethylsilyl propionate (TSP), as done originally, the metabolic profiling of NMR2 looked more like the other NMR than like the LCMS (online resource Fig. 3). Among the LCMS, one QTOF (Q2P) and one Orbitrap (O4P) shared the most common information with all the other instruments. NMR1 displayed the most ubiquitous information among the NMR instruments (Figs. 3a, 4b).

In the Test #2 network, LCMS and NMR were also located in two distinct regions (Fig. 3b). In this test, the QTOF Q1P and the NMR 4 shared the most common information with NMR and LCMS, respectively. QTOF Q4P shared the most common information with the other LCMS platforms.

Of note, for the LCMS instruments in both tests, the number of features retained as well as the LCMS technology (QTOF, TOF or Orbitrap) did not influence the proximity between instruments (Fig. 3).

### Effect of urine dilution in Test #1

In addition to the obvious differences between the spiked versus non-spiked urine samples, we addressed whether the interindividual differences in the metabolic profiling found in urine could result from variations in urine dilution. For this, a correction factor was calculated for each non-spiked sample based on the specific gravity method (Cone et al. 2009) (Online Table 5). For both NMR and LCMS, the total spectral intensity was used to recalculate a new CCSWA model after correction with the dilution factor (online resource Fig. 3). This model only emphasised the interindividual metabotype differences. It showed a very similar pattern of distribution before and after dilution correction among the individuals for the main related common components (CC2 panel A and CC1 panel B in Fig. 1 and in online resource Figure 4B, respectively). However, although individuals #5 and #8 were again identified as outliers, the normalisation of the data to the urine dilution factor also revealed another heterologous individual (#2, CC2 of online resource Figure 4, panel A) that was not previously found. Interestingly, this outlier was mainly detected by NMR (saliences for CC2, panel B of online resource Figure 4).

## Discussion

Our study was designed to evaluate the ability of untargeted metabolomics approaches to produce convergent results at the metabolic profiling level when performed on the same set of samples by instruments of various technologies and located in different laboratories using non-standardised procedures. This is to date the largest inter-laboratory test implemented for metabolomics.

The samples analysed were generated through two protocols purposely depicting a high and a low biological contrast situation and extracted from two biofluids, plasma and urine. These situations and matrices were thought to reflect the typical analytical metabolomics situations commonly encountered in human and animal studies. Although in Test #1 we spiked the samples with known standards, our main goal was not to make comparisons at the molecular level but to evaluate the inter-instrument convergence at the metabolic profiling level, which constitutes the bottom line of untargeted metabolomics.

This approach differed from previous attempts in which the instruments and/or the analytical conditions were in as near identical conditions as possible (Ward et al. 2010; Allwood et al. 2009; Viant et al. 2009; Benton et al. 2012) or possessed some variations authorised in post-acquisition procedures (Viant et al. 2009). Our test has the scope to help us assess to what extent results can be platform dependent and how trustworthy the findings may be when obtained under non-standardised conditions using instruments of different technologies.

The samples were analysed on relatively similar NMR instruments but on rather different LCMS systems, where the LC varied from conventional to very high pressure, was from different vendors, and used different chromatographic columns, ESI conditions, LCMS configurations (TOF, QTOF, orbital technology) and instrument series (see online resource). Additionally, specific spectral signal filtering and deconvolution methods were used by each participant. As a result of this instrumental, analytical, acquisition and post-processing heterogeneity, the signal generated by the various instruments could not be directly compared on a feature (variable extracted from the workflow) basis. Neither could they be compared on a metabolite basis, owing to metabolite annotation difficulties and discrepancies in instrument sensitivity. To circumvent this difficulty, we implemented spiking experiments in one of the two tests and also applied two statistical methods that allowed the comparison of the entire dataset generated by each platform rather than merely the individual features, namely, CCSWA and calculation of RV coefficients. The primary aim of this overall statistical analysis was to assess the convergence of the metabolic profiling obtained by several types of instruments and depicted as spectral information, regardless of the metabolite chemistry. In other words, we investigated how biological status, translated into metabolomics profiles, could be related when measured by instruments of various technological designs.

Our results also provided an overview of the number of features that could be extracted from the same set of samples compared with those that diverged dramatically across the platforms, both in Test #1 and Test #2, mainly due to post-processing methods. This is well highlighted in Test #1 by the number of discriminating features found by CCSWA related to the 32 standard molecules (Table 2). The RV coefficients examining the pair-wise relationships among the instruments also appeared unaffected by this factor in both tests, as exemplified in online resource Fig. 2 (average RV) and Fig. 3. Hence, redundancy or parsimony in the number of features extracted did not seem to compromise convergence in the information retrieved across the platforms. Participants used both PLS-DA models and VIP scores (Trygg et al. 2007) to select the discriminating features or used a standard *t* test. Compared to the selection made independently from CCSWA, the results were highly overlapping, indicating the consistency of the discriminating features isolated across platforms and the statistical methods used.

Not surprisingly, in the first test matching the native urine and the urine spiked with the standard mixtures, all the participants were able to discriminate the two groups quite clearly. This was not due to any dilution effect owing to the addition of the spiking solution since we carefully controlled this factor (see methods section). Of note, the addition of the standard mixture to the urine samples in Test #1 did not improve the metabolic profiling convergence (RV values) among either the NMR or the LCMS results (Table 3). This is likely because the amount of exogenous metabolites spiking the urines was constant among all the urine samples and thus did not contribute to the variation measured across the samples.

When the data were examined for NMR and LCMS separately, it appeared that, whereas NMR reported steady ‘within’ RV values (0.78) across both tests, this was not the case for LCMS, for which the RV values were lower in Test #2 versus Test #1 (Table 3). This cannot be ascribed to the lower sensitivity of LCMS in the low contrast situation (Test #2) because the addition of the standard mixture to the urine (Test #1), which artificially increased the contrast, did not result in a commensurate convergence among LCMS (Table 3). In fact, the addition of the standards did not improve the convergence among the NMR results, either. Compared to NMR, the lower RV values observed within LCMS in Test #2 (plasma samples) versus the RV of the non-spiked urine samples in Test #1 would suggest a matrix-related effect, in which LCMS was inferior to NMR in reporting the metabolic profiles in plasma. This could arise from the trace amount of proteins that remained in the plasma sample extracts, which would impair the LCMS analyses. This issue remains to be carefully addressed.

In Test #2, the vitamin D group could not be clearly distinguished from the untreated group, but the treated group displayed the greatest dispersion, suggesting individual differences in the treatment response. Additionally, in Test #2, a tendency for discrimination could be observed in the common component 2, in which the NMR instruments appeared more influential than did the LCMS instruments, as indicated by the CC2 scores. The same pattern also occurred for Test #1, in which the NMRs performed slightly better than the LCMSs in discriminating the groups (CC1 scores and loadings of Fig. 1). It should be noted that for sensitivity reasons, the standard concentration was 10-fold higher in the urine to be analysed by NMR. On the other hand, the individual metabotypes were more repeatedly reproduced across the LCMS instruments than they were in NMR, at least in the Test #1 urine samples (scores and loadings CC2 in Fig. 1a, b, low dispersion along the inter-individual CC2 in online resource Figure 5A). This is further outlined by the better performance of LCMS in detecting the two outliers in the Test #1 urine and especially in detecting the blind outlier. For the latter, most of the LCMS instruments found estroprogestative hormone derivatives and related steroid hormone derivatives as the leading discriminating factors, as could be expected according to the physiological situation of that individual (a mid-term pregnant woman). NMR did not report similar discriminating compounds but rather compounds such as alanine, threonine, lactate, and glycine, which are difficult to relate specifically to the particular physiological status of the outlier. This might be due to differences in sensitivity, as the concentrations of estroprogestative derivatives in pregnant women are reported to range from 3 to 5 µmol/L urine (0.5 to 0.8 µg/mL) (Johnson and Williams 2004), which is within the lowest level or slightly below the limit of detection for NMR. In addition, possible overlap with other signals could impair detection. Also likely is that the compounds found to be discriminating by NMR could be so for LCMS as well, but to a lesser extent than the estroprogestative derivatives primarily detected by LCMS. They could thus be excluded from the list of the features retained at the high correlation threshold level chosen (*r* > 0.8 for LCMS). Also interestingly, some laboratories used automated annotation from METLIN for the XCMS output, which gave either multiple hits for each m/*z* feature and/or irrelevant annotation in the current biological context (see Q6, O1, and most obviously O4 in online resource Table 4). This is a good illustration of the care that should be implemented in post-data acquisition curation.

For confidentiality reasons, the discriminating metabolites were not investigated for the unexpected outlier. Interestingly though, LCMS also generally performed better than did NMR in identifying this individual, except for one NMR, which similarly pinpointed this individual (common component 3 of Fig. 1b). This NMR additionally distinguished itself from the other NMRs by clustering with LCMS for the other characteristics, such as inter-individual metabotypes description or group discrimination in Test #1 (common component 2 and common component 1 in Fig. 1b, respectively). As a result, it also more closely clustered with all LCMS in the network analysis (Fig. 3a). The only noticeable difference with the other NMR platforms was that normalisation was performed on trimethylsilyl propionate (TSP), whereas the other methods used the total peak area. In general, normalisation is performed on the total spectral area when dealing with NMR urine analysis to prevent bias due to the dilution factor varying widely between urine samples. This is less critical in plasma due to the much tighter regulation of metabolite contents. In fact, when NMR2 data were normalised to the total intensity area as done for the other NMR, its specificity vanished and it performed closer to the other NMR for its average RV values, CCSWA scores (except for individual #8 detection) and RV closeness in network analysis (online resource Figure 3).

Thus, although obvious differences owing to technological designs occurred between LCMS and NMR that may lead to individual metabolite mismatching, the convergence in the spectral characteristics extracted by either LCMS or NMR was generally satisfactory in our studies and could even be improved by adjusting the data post-processing normalisation of an internal standard, such as TSP. We also assessed whether different data scaling would affect the results, but found this factor to be irrelevant (not shown). Additionally, when focusing on the LCMS systems, none differed according to whether the instrument configuration was based on time-of-flight or orbital design, ionisation mode (positive/negative) or various LC systems. This result extends to multiple instruments the findings on metabolic profiling made by others comparing only two mass-analysers (Gika et al. 2010; Glauser et al. 2013). However, one LCMS Orbitrap (O3) was somewhat separated from the others in Test #2 when run in positive mode but not in negative mode. This was not observed in Test #1, thus indicating a poor contextual acquisition due to an unidentified and non-constitutive reason (e.g., source contamination). One instrument (Q6) in Test #1 was also shifted away from the others, as illustrated in the network display (Fig. 3a), but this was corrected when the XCMS parameters were tuned differently and run on the same original dataset (not shown). With regard to this post-processing parameter, both the QTOF Q2 and the Orbitrap O4 operated in positive mode shared the most common information with all the other platforms in Test #1, whether LCMS or NMR. O4 in particular, although similar to O3, used the default XCMS conditions embedded in MetaboAnalyst (http://www.metaboanalyst.ca/) and displayed somewhat more robust relationships with the other instruments. Nonetheless, at this stage we cannot recommend XCMS specific parameters to be tuned to improve LCMS profiling because a specific design is required for such an objective (Smith et al. 2006). Of note is that others found this deconvolution step weakly critical, even when performed with different software and algorithms (Gürdeniz et al. 2012).

Due to its robustness, the application of untargeted metabolomics to epidemiological studies has been restricted thus far to NMR (Holmes et al. 2008), although methods have been implemented for large series for LC–MS (Dunn et al. 2011). However, these latter could suffer from mathematical bias. Conversely, LCMS is more sensitive and produced data of higher information density. The good convergence we found in the spectral information extracted across LCMS and NMR also indicated that, although the analytes to be detected may be individually different, they all described the same biological status. This is a further illustration that metabotypes are defined over the whole metabolome, whose constituents are in close equilibrium. Thus, an interesting strategy to analyse large sets of samples would be to perform a screening using NMR to select sub-groups of interest for which the metabolomics coverage could be completed by LCMS.

## Conclusion

Our primary aim was to assess the disparity of untargeted metabolomics approaches in characterising the same metabotypes translated into different spectral datasets collected from instruments of various technologies under unstandardised conditions. Our main finding is that there is a high convergence in the spectral information produced from the various instruments to describe the same set of samples, irrespective of the type of standardisation, deconvolution method, LCMS analyser or configuration (QTOF, TOF, Orbitrap delivered by various LC systems, ionisation modes). The performance of instruments and methods to identify and match individual metabolites, especially using LCMS systems (Benton et al. 2012), remains to be explored in greater depth (such as in http://www.abrf.org/index.cfm/group.show/MetabolomicsResearchGroup(MPRG).60.htm). Additionally, including GCMS in such interlaboratory tests for comparison with other analysers appears necessary. At last, our results suggest that data fusion across platforms using either similar or different analytical methodologies and operated with a variety of experimental settings is possible. Owing to these differences of experimental settings, high level data fusion relying on identified metabolites or annotated features should be the most relevant approach. In this context, sharing common quality control samples dedicated to a given biological matrix should permit cross platform normalisation (Dunn et al. 2011) and could thus be an efficient way to achieve this goal. Finally, in addition to the analytical convergence, the absolute validation of untargeted metabolomics in a ring test would also rely on the convergence of the biological outcomes arising from the analyses. This remains to be investigated.

## Electronic supplementary material

Below is the link to the electronic supplementary material.

Supplementary material 1 (DOCX 587 kb)
