# Supplementary material for: Can we trust untargeted metabolomics? Results of the metabo-ring initiative, a large-scale, multi-instrument inter-laboratory study
Source: Metabolomics. 2014 Oct 14;11(4):807–21. doi: 10.1007/s11306-014-0740-0 (PMC4475541; doi:10.1007/s11306-014-0740-0)
Supplement: Supplementary file 1 — Supplementary material 1 (DOCX 587 kb) [file 11306_2014_740_MOESM1_ESM.docx]

**Online resource methods**

**Can We Trust Untargeted Metabolomics?: Results of the Metabo-Ring initiative, a large-scale, multi-instrument inter-laboratory study**

**Sample collection and preparation**

- *Test #1*

For 20 hours before sample collection, volunteers were asked to avoid alcohol and medication and to moderate their physical activity. They were instructed to eat a dinner between 7 and 9 pm comprising 2 regular slices of ham, approximately 100 g dry weight of spaghetti with 20 g of butter, and one plain yoghurt with 20 g of refined sugar, accompanied by tap water only. The volunteers were asked to fast until urine collection on the following morning. In the morning, the totality of the bladder was emptied, and the second void was collected in the laboratory in 50 mL Falcon tubes with a screw cap.

The added mixture of 32 standard molecules contained (online resource Table 1) ascorbic acid, citrulline, creatinine, taurine, uric acid, caffeine, glutaric acid, inosine, isoleucine, leucine, pyroglutamic acid, methionine, methylmalonic acid, N-methylhistidine, aminobenzoic acid, phenylalanine, proline, riboflavin, adenine, adenosine, adipic acid, azelaic acid, caffeic acid, tryptophan, tyrosine, uracil, uridine, chenodeoxycholic acid, cholic acid, cortisone, deoxycholic acid, and glycocholic acid. A stock solution was prepared containing all the compounds, each at a final concentration of 0.1 mg/mL. For LCMS analysis, 100 µL of the stock solution was evaporated to dryness under a flux of nitrogen and resuspended with 100 µL of the urine sample. The mixture was centrifuged at 14000 RPM for 10 min at 4°C, and 50 µL of the supernatant was removed and diluted with 150 µL of extrapure water (MilliQ). After centrifugation at 3000 RPM for 10 min at 4°C, an aliquot of the supernatant was transferred to an LC vial for LC-MS analysis. For NMR analysis, 1.5 mL of the standard mix stock solution was dried and rediluted with 500 µL of urine. Prior to analysis, 200 µL of phosphate buffer was added to each of the urine samples (phosphate buffer (pH 7.4): Dissolve 2.885 g anhydrous Na_2_HPO_4_, 0.525 g anhydrous NaH_2_PO_4_, 17.2 mg sodium 3-trimethylsilylpropionate-*d*_4_ (TSP), 60 mg sodium azide (NaN_3_) in 100 mL H_2_O/D_2_O (80%/20% vol/vol), *see* Beckonert *et al*, Nature Protocols, 2007, 2, 11, 2692-2703), and the mixture was centrifuged at 14000 RPM and 4°C for 10 min. Five hundred mL of the supernatant was then transferred to an NMR tube. A pre-study using various concentrations of the standard mixture allowed the procedures to be chosen to enable optimal detection, with the concentrations of each compound brought to 25 µg/mL, and 250 µg/mL in the urine samples to be analysed by LCMS and NMR, respectively. A quality control (QC) sample for either the spiked or non-spiked urine and a blank sample were also prepared by pipetting and merging 50 µL of each respective urine sample or by replacing the urine with extrapure water (blank), respectively.

All the samples were prepared in one laboratory and dispatched to all the participants in dry ice. The samples were stored at -80°C until analysis within 1-3 months. Two vials per sample were provided for duplicate analysis.

The urine density was also measured to apply a correction factor whenever necessary (see experimental section in the manuscript and online resource Table 5).

In addition, as specified in the experimental section, an outlier (pregnant woman at mid-gestation) was blindly introduced, and the participants were asked to seek for an outlier individual and to identify and annotate the discriminating features; these are reported in the following online resource Table 4.

|  |  |  |  |  |  |  |
| --- | --- | --- | --- | --- | --- | --- |

- Test #2: Diet composition

**see online resource Table 2**

- *Statistics*

*RV coefficients*

The RV coefficient between the two data tables X1 and X2 was calculated as follows:

$RV(X1,X2)=trace(W1*W2)/(\sqrt{trace(W1*W1)}*\sqrt{trace(W2*W2)})$

Where:

W1=X_1_*X_1_^T^

W2=X_2_*X_2_ ^T^

t_W1 W1=trace(W1*W1)

t_W2 W2=trace(W2*W2)

t_W1 W2=trace(W1*W2)

The RV coefficients matrix was calculated based on the 22 data tables obtained from Test #1 and on the 12 data tables obtained from Test #2. For each Test, the RV coefficients indicated the strength of the association between the data tables. For each data Table, the average RV coefficient was calculated as the average of all the RV coefficients between this Table and all other tables. A value close to one indicates that the corresponding Table contains similar information to all the others. To test the significance of the assessment of the RV coefficients, the coefficients were re-calculated after 100 random re-samplings. For each of these, the observations (*i.e.*, human urine samples for Test #1 and plasma rat samples for Test #2) were ranked in a random order independently in all data tables. Therefore, the assumption was that the RV coefficients calculated on randomly ordered samples would be close to zero and much lower than the RV coefficients calculated on the original data. For each instrument, the distribution of the 100 random RV coefficients was graphically represented using boxplots (Figure 1). The average RV coefficients within the NMR and LCMS instruments were also compared.

*Common components and specific weights analysis (CCSWA or ComDim)*

The aim of CCSWA is to investigate the relationships among several data tables. Derived from the consensus or global scores of all the tables, T(:,i), local scores were calculated for each Table, j, and each Common Component, i, by the following procedure:

Local Loadings, LocalLx{j}(i,:), were calculated for each centred and normalised data Table, **Table{j}**, and each Common Component, *i*, as:

LocalLx{j}(i,:) = inv( T(:,i)' * T(:,i) ) * T(:,i)' * Table{j};

For common component 1, Table {j} is used as is; for subsequent common components, it is deflated first.

These Local Loadings were then used to calculate the corresponding Local Scores, **Local_T(j,:,i)**

Temp = ( LocalLx{j} * Table{j}' )';

Local_T(j,:,i) = Temp(:,i) / norm( Temp(:,i) );

A local score represents the projection of individuals (samples) in the common space for a given data Table. By projecting the samples using both global scores and local scores onto the same plot, the dispersion of all the data tables around the consensus scores in the common space can be visualised.

*Identification of discriminating features based on the CCSW analysis in Test #1*

One of the common components calculated by the CCSW analysis gave complete separation between the non-spiked group and the spiked group. Given the experimental setup, this common component should explain the maximum amount of common variance shared by all instruments (*e.g.*, common spectral information). Thus, the CCSW analysis calculated scores for all samples that represented the common component. For each data Table, correlations between the common component and the features were calculated. Features with a correlation greater than 0.8 were considered as discriminating features. The number of discriminating features selected using the CCSW analysis as well as the list of these features were matched and compared with the discriminating features selected independently by platform operators.

*Scaling*

Each data Table was scaled independently to provide comparable data between instruments. Because NMR and LCMS instruments provide data with different characteristics, two different scalings were applied. The rationale was to standardise each data Table in order to retain the specific structure of each method while avoiding particular tables that had an effect because they were similar due to data size. The data extracted from the NMR instruments were Pareto-scaled ^14^. The data produced by the LCMS instruments were first Log10 transformed after the addition of a constant to avoid negative values. Then, the Log10 transformed data were Pareto-scaled.

**Data acquisition and post-processing**

- *Participant N1*

Urine samples were prepared by mixing urine (500 µl) with phosphate buffer (200 µl, pH 7.4, 0.2 M) containing 10% D_2_O as a field frequency lock and sodium trimethylsilyl-2,2,3,3-tetradeuteropropionate (TSP, internal standard) as a chemical shift reference. Buffered urine samples were then centrifuged at 13,000 g for 10 min to remove any precipitates, and aliquots of 0.6 mL were transferred to 5 mm NMR tubes for ^1^H NMR analysis.

All ^1^H NMR spectra were acquired at 300 K on a Bruker DRX-600 Avance NMR spectrometer operating at 600.13 MHz for ^1^H resonance frequency using an inverse detection 5 mm ^1^H-^13^C-^15^N cryoprobe. The spectra were collected using a solvent suppression pulse sequence based on a 1D nuclear Overhauser effect spectroscopy pulse sequence (T_rd_-90°-t_1_-90°-t_m_-90°-T_aq_) to saturate the residual water signal, first during the relaxation delay (T_rd_=2s) and second during the mixing time (t_m_=150 ms). The delay t_1_ was held constant at 3 μs. For each 1D NOESY spectrum, 128 free induction decay values (FIDs) were collected into 32,768 data points using a spectral width of 12,000 Hz with an acquisition time of 1.36 s. For all the spectra, the FIDs were multiplied by an exponential weighting function corresponding to a line broadening of 0.3Hz and zero-filled before Fourier transformation. The acquired NMR spectra were phase and baseline corrected manually and referenced to the TSP signal (δ=0 ppm).

The urine spectra were data-reduced prior to statistical analysis using AMIX software (Analysis of Mixtures, Bruker, v3.9.11, Karlsruhe, Germany). The spectral region δ 0.5-10.0 ppm was segmented into consecutive non-overlapping regions of 0.01 ppm (buckets). The region δ 4.5-6.5 ppm in the urine spectra was excluded from the statistical analysis to eliminate artefacts from residual water and urea resonances. To minimise possible differences in concentration between the samples, each integrated region was normalised to the total spectral area. The values of all variables were Pareto-scaled before PCA (Principal Component Analysis) and PLS-DA (Partial Least Squares Discriminant Analysis) analyses using SIMCA-P software (version 12, Umetrics, Umea, Sweden). Discriminant variables were determined using VIP (Variable Importance in the Projection), and an arbitrary threshold of VIP>1.5 was chosen to select the variables. The spectral assignments were based on matching the 1D and 2D data to reference spectra in a database (<http://brmb.wisc.edu/metabolomics>; <http://www.hmdb.ca/>) and to reports in the literature.

- *Participant N2*

Sample preparation:

Phosphate buffer (pH 7.4) was prepared from anhydrous sodium phosphate dibasic (2.885 g), anhydrous sodium phosphate monobasic (0.525 g), sodium 3-trimethylsilylpropionate-d4 (17.2 mg), and sodium azide (60 mg) dissolved in 100 mL of a mixture of water and deuterium oxide (80% :20%; vol : vol). The phosphate buffer solution (200 μL) was added to urine (500 μL), homogenised by vigorous shaking, and centrifuged at 20000 g and 4°C for 10 min. The supernatant (600 μL) was transferred to an NMR tube.

Spectroscopic analysis:

NMR spectra were acquired on a Bruker AVANCE III 600 spectrometer (Magnet system 14.09 T 600 MHz/54 mm operating at 600.17 MHz for 1H) using a TXI 5 mm z-gradients probe and running the TOPSPIN 2.1 (Bruker) software. Automation was controlled by the IconNMR software. The sample temperature was regulated at 300 K with an allowance of approximately 5 min for setting the temperature before data acquisition. Automatic tuning and matching of the probe was carried out for each sample. The samples were not spun. Standard shim settings from a reference file were loaded with each new sample. D_2_O (from buffer) was used for the field/frequency lock and shims were optimised for each sample using the Topshim programme for gradient shimming (Z1 to Z6) followed by trimming of (Z1, Z2, X, Y) the shims. The 90^0^ pulse length was determined for the first sample and applied to all subsequent samples.

For each sample 1D proton with water suppression, pulse sequence (NOESY 1D) and 2D proton 1H (JRES) were performed ^[^[^1^](#_ENREF_1)^]^. The sequence for the NOESY 1D repeat was –D1-t-90°-t-90°-tm-90°-AQ, where D1 (4 s) is the relaxation delay, 90° is the already determined 90° radio-frequency pulse length, t (4 µs) is a very short delay, tm (0.15 s) is a mixing time delay and AQ (3.90 s) is the data acquisition time. Low power rf irradiation was applied at the water frequency during D1 and tm to pre-saturate the water signal. Spectra were run with a fixed receiver gain (RG=64). Each spectrum consisted of 128 scans and 8 dummy scans of 64K data points with a spectral width of 14 ppm. Furthermore, two-dimensional J-resolved NMR spectra were acquired. The pulse sequence comprises relaxation delay—90°—(t1/4)—180°—(t1/2)—180°—(t1/4)—acquisition, where t1 is an incremented time delay used to create an indirect time axis for the second dimension. 2D 1H JRES NMR spectra were acquired with a 2.0 s relaxation delay using 4 transients per 64 increments that were collected into 32k data points, using spectral widths of 8417.5 Hz along the direct dimension (*i.e.*, chemical shift axis) and of 80 Hz along the indirect dimension (*i.e.*, spin–spin coupling axis) and using excitation sculpting for the water suppression.

On samples (K1, N11, N5), HSQC, 2D H-1/X correlation *via* double inept transfer using sensitivity improvement, phase sensitive using Echo/Antiecho-TPPI gradient selection with decoupling during acquisition using trim pulses in inept transfer using shaped pulses for all 180 degree pulses on f2 – channel with gradients in back-inept spectra were recorded. The spectra were acquired with a 1.5 s relaxation delay using 32 scans for K1 and 128 scans for N1 and N5 per 256 increments that were collected into 2K data points using spectral widths of 8417.5 Hz in F2 and 30185.3 Hz in F1. All 2D spectra were phased and calibrated at 0.0 ppm to TMSP.

Data analysis:

The time domain signals obtained with the NOESY 1D sequence were Fourier transformed with a 0.3 Hz exponential line broadening factor zero filled to give spectra with 64K real points, phase corrected, baseline corrected (usually only the first two terms of a 5th order polynomial correction were used) and, for urine, referenced to TSP at 0 ppm. Prior to Fourier transformation, where stated, JRES spectra were zero filled to give spectra with 32K real points in the direct dimension and 64K real points in the indirect dimension. Data in the direct and then indirect dimensions were either apodised using SINE apodisation (*i.e.*, multiplied by a sine-bell window function in each dimension) and SEM apodisation (*i.e.*, multiplied by a combined exponential and sine-bell function along the direct dimension and by a sine-bell function along the indirect dimension). Then, the 2D spectra were tilted by 45° and symmetrised. Finally, each spectrum was calibrated by setting TMSP to 0 ppm on the direct and indirect dimensions, respectively. Positive skyline projections of the 2D spectrum along F2 were made. A 1D spectrum (pJRES) was obtained.

The phased, baseline-corrected and referenced 1H NMR spectra (NOESY 1D and pJRES) were automatically reduced to ASCII files using AMIX (Bruker). The spectral intensities (positive) were scaled to TMSP and reduced to integrate regions or ‘‘buckets’’ of equal width (0.04 ppm) corresponding to the region of d 10.5 to δ 0.0. The region from 4.85 ppm to 4.75 ppm was removed from the analysis due to the residual signal of water. Proton signals corresponding to TSP-d4 (at δ 0.00 ppm) were also removed. A total of 252 buckets were created over the spectral range. The generated ASCII file was imported into Microsoft Excel (Microsoft Corporation) for the addition of labels. Principal component analysis (PCA) and partial least squares discriminant analysis (PLS-DA) were performed with SIMCA-P software (v. 12.0, Umetrics, Umea, Sweden). The scaling method for PCA was Pareto and was unit of variance for PLS-DA. To test the significance of the differences observed between the spiked and control samples, a Mann-Whitney-Wilcoxon test using R software (http://www.r-project.org/) was applied.

Identification of the different metabolites was obtained *via* data comparison with NMR spectra (Amix database pH 7) and with standard metabolite chemical shift tables from the HMDB database.

Reference:

1. Ludwig C, Viant MR: Two-dimensional J-resolved NMR spectroscopy: review of a key methodology in the metabolomics toolbox. *Phytochemical analysis: PCA* 2010, 21(1):22-32.

- *Participant N3*

NMR samples were prepared using 200 μl of pH 7.4 200 mM phosphate buffer (H2O/D2O, 80/20 v/v) mixed with 400 μl of urine and placed into a 5 mm NMR tube. All the 1D ^1^H NMR experiments were carried out on a Bruker Avance spectrometer operating at 600 MHz for the ^1^H frequency using a 5-mm broadband observe probe at 300 K. Spectra were collected using a solvent suppression pulse sequence based on a 1D nuclear Overhauser effect spectroscopy pulse sequence (T_rd_-90°-t_1_-90°-t_m_-90°-T_aq_) to saturate the residual water signal, first during the relaxation delay (T_rd_=2s) and second during the mixing time (t_m_=150 ms). The delay t_1_ was held constant at 3 μs. For each 1D NOESY spectrum, 128 free induction decay values (FIDs) were collected into 32,768 data points using a spectral width of 9,600 Hz with an acquisition time of 1.71 s. For all the spectra, the FIDs were multiplied by an exponential weighting function corresponding to a line broadening of 0.3 Hz and zero-filled before Fourier transformation. The acquired NMR spectra were phase and baseline corrected manually and referenced to the taurine signal (δ=3.43 ppm).

Before statistical analysis, the spectral region between 0.5 and 10.5 ppm was divided into buckets with variable sizes (one bucket per identified signal when possible; alternatively, 0.04 ppm-width buckets were used) using AMIX software (Bruker Biospin, Karlsruhe, Germany) in order to reduce the residual shifts of the signals due to pH variations. The region δ 4.77-6.56 ppm in the urine spectra was excluded from the statistical analysis to eliminate artefacts from residual water and urea resonances. To minimise possible differences in concentration between the samples, each integrated region was normalised to the total spectral area.

Principal component analysis based on the correlation matrix was carried out on the NMR dataset using SIMCA P+ v12 software (Umetrics, Umea, Sweden). Buckets with a PC1 coefficient superior to 0.5 were considered as statistically significant for discrimination between the 2 analysed groups.

Assignments were performed using Chenomx software (Chenomx Inc., Canada), 1H-1H TOCSY and 1H-13C HSQC experiments.

- *Participant N4*

NMR procedure

The urine samples were thawed at room temperature and prepared for ^1^H NMR spectroscopy by mixing 400 *μ*L urine with 200 *μ*L phosphate buffer (0.2 M Na_2_HPO_4_ and 0.038 M NaH_2_PO_4_, pH 7.4) prepared with 80% D_2_O and containing 9 mM sodium azide and 1 mM TSP (3-trimethylsilyl propionate) as a chemical shift reference. The sample was shaken, and 500 *μ*L was transferred into a 5 mm NMR tube for spectral acquisition. ^1^H NMR spectra were recorded at 600 MHz on a Bruker Avance spectrometer (Bruker BioSpin GmbH, Rheinstetten, Germany) running TOPSPIN 2.0 software and fitted with a cryoprobe and a 60 slot autosampler. The acquisition order was randomised with respect to the collection date and sample codes. Each ^1^H NMR spectrum was acquired with 128 scans, 90° pulses (10.8 *µ*s), a spectral width of 8389 Hz, an acquisition time of 1.95 s and a relaxation delay of 2.0 s. The *noesygppr1d* pre-saturation sequence was used to suppress the residual water signal with low power selective irradiation at the water frequency during the relaxation delay and mixing time (0.15 s). The spectra were transformed with zero filling and 1 Hz line broadening, manually phased, baseline corrected and referenced by setting the TSP methyl signal to 0 ppm. Metabolites were identified using information found in the literature [1] or on the web (Human Metabolome Database http://www.hmdb.ca/) and by using the 2D-NMR methods COSY, HSQC, and HMBC.

NMR data analysis

The NMR Spectra were prepared for statistical analysis using the Bruker AMIX software v3.9. The spectra were divided along the horizontal axis into variable width “buckets” (or bins) using the AMIX graphical editor to draw buckets that included, where possible, recognisably complete individual peaks or multiplets. The intensities within each bucket were summed and divided by the bucket width, and the bucket intensities were normalised to the same total intensity for each sample to give the final bucket Table. Regions with only background noise and the water resonance were not included in the buckets. Multivariate statistical analysis (PCA and PLS-DA) was carried out using the PLS Toolbox v5.5 (Eigenvector Research Inc., Wenatchee, Washington, USA) running within Matlab® v7.6 (MathWorks Inc., Massachusetts, USA).

[1] Nicholson, J.K., Foxall, P.J.D., Spraul, M., Farrant, R.D. et al. 750 MHz ^1^H and ^1^H-^13^C NMR Spectroscopy of Human Blood Plasma. *Anal. Chem.* 1995, *67,* 793-811.

- *Participant N5*

^1^H NMR spectroscopy

A volume of 0.5 mL of urine was placed into a 5-mm diameter specific tube together with 0.1 mL of D_2_O. The proton spectra were acquired on a Bruker 500 Avance III NMR spectrometer at 25°C. A signal was acquired after a 90° pulse of 32K data points on a spectral window of 5000 Hz. The relaxation delay was 4 s. The water signal was suppressed by a pre-saturation sequence using low-power irradiation (0.03 W for 2 s) on the water-signal frequency during the relaxation delay. The resulting free induction decays obtained with 128 transients were processed by NmrPipe software. A Fourier transformation was applied with an exponential window function to produce a 1-Hz broadening line. The spectra were phased, and a spline baseline correction was applied with two points at 0 and 9 ppm. Each spectrum was referenced using the lactate signal (1.32 ppm). The spectral region between 0–9 ppm was divided into 9000 spectral regions 0.001 ppm in width, called buckets, using a personal C program. The water regions were excluded. Each bucket was integrated and scaled to the total summed integrals for each spectrum.

Multivariate analyses on NMR data

After processing the spectra, statistical analyses were performed using in-house Matlab® code. PCA was first performed to detect any group separation based on NMR signal variability. This method also enabled the detection of any excluded outliers, defined as observations situated outside the 95% confidence region of the model.

An orthogonal projection to latent-structure (OPLS) analysis was run to discriminate the two groups. Variables with VIP > 1.5 were considered as discriminant.

- *Participant Q1*

The samples were analysed on an Agilent 1200 RRLC coupled to a Bruker microTOF ESI-hybrid quadrupole-time of flight mass spectrometer (Wissembourg, France). The liquid chromatographic conditions were: autosampler set at 4°C; column, EC 100/2 Nucleodur C18 pyramid; particle size 1.8 μm (Macherey-Nagel, Les Ulis, France); operated at 40°C; started from solvent A (95% water, 5% acetonitrile, 0.1% formic acid) to solvent B (95% acetonitrile, 5% water, 0.1% formic acid) from 0–10 min at 0.4 mL/min in a 40°C column oven; thereafter switched to 95% A and 5% B for 6 min, held 2 min, and then returned to 100% A for 5 min. Prior to analysis, the time of flight analyser was calibrated with a sodium formate solution (*m/z* range from 91 to 1122, HPC and quadratic algorithm, 32 m/z used, over 90% accuracy at 0.001% mass window), and calibration was automatically performed at the beginning of each analysis. The mass spectrometry (MS) conditions were as follows: mass spectrum mode from m/z 50 to m/z 1500; capillary kV, 4.5; capillary temperature, 250°C; cone voltage 40 V; drying gas flow set at 9.5 L min-1 and nebulising gas pressure (nitrogen) at 2.4 bar; positive or negative electrospray ionisation mode (ESI+ or ESI-).

*Mass-spectrometry data processing*

Raw data files were converted into netCDF format prior to deconvolution with XCMS in the R environment. The XCMS parameters were (default parameters when not stated):

xset<-xcmsSet(method="centWave", peakwidth=c(3,15), snthresh=5)

xset2<-retcor(xset, method="obiwarp", profStep=0.1, plottype="deviation")

xset2<- group(xset2, bw=5, mzwid=0.025)

The Centwave algorithm was used for feature detection, and obi-warp was used for retention time peak alignment. Unreliable peaks were removed using a coefficient of variation cut off ≤ 20% in the quality control samples and applied to all samples. Further peak redundancy removal (de-isotoping and de-adduction) was carried out by auto-correlating the intensity feature values across the quality control samples into the pc group windows calculated by the CAMERA open source package, identifying adducts and isotopes occurring from the parent molecular ions. This was achieved using an in-house script run in the R environment.

The mass spectrometry features retained after statistical analysis were then converted into elemental chemical formulas using Data Analysis (Bruker Daltonics, Bremen, Germany) based on their exact mass at an approximately 10000 mass resolving power, their isotopic pattern (sigma-fit cut off ≤ 20 at Δppm ≤ 15) and the adducts formed. The matching of elemental chemical formulas to molecular structure was performed through the web interface MZedDB, allowing simultaneous database repository requests.

Statistical analysis was performed using SIMCA P+ v12 (Umea, Sweden). Calculations were performed on log10-transformed and Pareto-scaled data. Principal component analysis of the pooled data with the quality control samples revealed that the analytical variance was satisfactorily low and much lower than the biological variance. All the models evaluated were tested for over fitting. To select which spectral features were significant in the PLS analysis, we clustered the loading scores, associating the w* (X loadings) with the c (Y loadings) using two-dimensional hierarchical clustering analysis (HCA). The clusters tightly associating the w* and the c were visualised on the loading plot of the PLSDA analysis. The most discriminant features were selected using the VIP algorithm of the SIMCA software, with the VIP values selected corresponding to the boundaries of the "w*c " clusters calculated from HCA (here, VIP values ≥ 1.35).

- *Participant Q2*

The samples were analysed on an Ultimate 3000 (Dionex, Sunnyvale, USA) pump coupled to a Bruker Impact TOF ESI-hybrid quadrupole-time of flight mass spectrometer (Wissembourg, France). The liquid chromatographic conditions were: autosampler set at 4°C; column, EC 100/2 Nucleodur C18 pyramid; particle size 1.8 μm (Macherey-Nagel, Les Ulis, France); operated at 40°C; started from solvent A (95% water, 5% acetonitrile, 0.1% formic acid) to solvent B (95% acetonitrile, 5% water, 0.1% formic acid) from 0–10 min at 0.4 mL/min in a 40°C column oven; thereafter switched to 95% A and 5% B for 6 min, held 2 min, and then returned to 100% A for 5 min. Prior to analysis, the time of flight analyser was calibrated with a sodium formate solution (*m/z* range from 91 to 1122, HPC and quadratric algorithm, 32 masses used, over 90% accuracy at 0.001% mass window), and calibration was automatically performed at the beginning of each analysis. The mass spectrometry (MS) conditions were as follows: mass spectrum mode from m/z 50 to m/z 1500; capillary kV, 4.5; capillary temperature, 250°C; cone voltage 40 V; drying gas flow set at 9.5 L min-1 and nebulising gas pressure (nitrogen) at 2.4 bar; positive electrospray ionisation mode (ESI+).

*Mass-spectrometry data processing*

Raw data files were converted into netCDF format prior to deconvolution with XCMS in the R environment. The XCMS parameters were (default parameters when not stated):

xset<-xcmsSet(method="centWave", ppm=5, peakwidth=c(3,15), snthresh=5)

xset2<-retcor(xset, method="obiwarp", profStep=0.1, plottype="deviation")

xset2<- group(xset2, bw=5, mzwid=0.01)

The Centwave algorithm was used for feature detection, and obi-warp was used for retention time peak alignment. Unreliable peaks were removed using a coefficient of variation cut off ≤ 20% in the quality control samples and applied to all samples. Further peak redundancy removal (de-isotoping and de-adduction) was carried out by auto-correlating the intensity feature values across the quality control samples into the pc group windows calculated by the CAMERA open source package, identifying adducts and isotopes occurring from the charged molecular species.

The mass spectrometry features retained after statistical analysis were then converted into elemental chemical formulas using Data Analysis (Bruker Daltonics, Bremen, Germany) based on their accurate mass at a mass resolving power of approximately 40000, their isotopic pattern (sigma-fit cut off ≤ 20 at Δppm ≤ 15) and the adducts formed. The matching of the elemental chemical formulas to their molecular structure was performed through the web interface MZedDB, allowing simultaneous database repository requests.

Statistics were performed as for partner Q1.

- *Participant Q3*

Chromatography was performed using the Waters Acquity UPLC module. Six µL of diluted half urine samples were injected into a 100 x 2.1 mm 1.7 µm BEH Shield RP18 column (Waters) at 30°C. The mobile phase components were A: H_2_O with 1% formic acid and B: acetonitrile with 1% formic acid. The column was eluted with a gradient of 100% A held for 2 min, followed by a decrease to 90% A over 2-7 min, and then to 5% A over 7-22 min. The mobile phase was then returned to 100% A at 22.1 min for a 4-min length re-equilibration. The flow rate was set to 0.4 mL/min. The Waters QToF-Micro source temperature was set to 120°C with a cone gas flow of 40 L/h, a desolvation temperature of 330°C, and a nebulisation gas flow of 650 L/h. The capillary voltage was set to 3,000 V, and the cone voltage was set to 30 V. The mass spectrometry data were collected in the continuum mass spectrum mode with a scan range from 70 to 1,000 m/z in positive ion mode.

All analyses were acquired using the lockspray with a frequency of 5 s to ensure accuracy. Leucine enkephalin was used as a lock mass compound. The analytical sequence was randomised. The stability of the analytical system was monitored using pooled samples injected one time at the beginning of each sequence and then again after each set of ten samples.

*Mass-spectrometry data processing*

Raw data files were converted into NetCDF format prior being extracted and aligned using XCMS. Feature extraction was performed with matchfilter. The XCMS parameters were: *fwhm: 10; sn: 1; step: 0.1; steps: 2; minsamp: 2; mzwid: 0.1* and *bw: 10; correction method: loess; intensity: peak height.* The CAMERA script for adduct and isotope identification was used.

*Statistics*

ANOVA was used first to determine the significant features at *P* < 0.05. Additional PCA and PLSDA was performed using SIMCA P11+ (Umea, Sweden). Significant features were selected according to their respective VIP values > 1 calculated from the SIMCA software. PLSDA model validation was performed by a permutation test.

- *Participant Q4*

Liquid chromatography

Chromatographic separation was performed with an Ultimate 3000 (Dionex, Sunnyvale, USA) pump on a reversed phase Uptisphere Strategy C18 NEC column (2.1 mm x 100 mm, 2.2 µm particle size, Interchim). The analytes were eluted by a 25-min gradient, which started at 100% A (Water + 0.1% formic acid) for 2 minutes, changed to 100% B (acetonitrile + 0.1% formic acid) over 18 min, maintained at 100% B for 5 min, and then returned to the initial condition for equilibration over 2 min. The flow rate was 0.25 mL min^-1^, and the column temperature was 25°C. The autosampler was set at 4°C for the duration of the analysis. Five µL of samples were injected.

ESI-HRMS

High-resolution mass fingerprints were acquired on a quadrupole - time of flight analyser (MicroToF Q II, Bruker, Bremen, Germany) in positive and negative ionisation modes. The spectrometer parameters corresponding to the capillary voltage, capillary temperature, nebuliser gas flow and dry gas flow were set as follows: -4.5 kV and 3.8 kV, 180°C, 2.4 bar, and 8 L min^-1^, respectively. External mass calibration of the instrument was performed using a solution of salt of lithium at the beginning of the chromatographic gradient using a divert valve and a separate pump. The mass accuracy of the m/z calibration standard was below 3 ppm in the positive mode and below 2 ppm in the negative mode. Centroid mass spectra were acquired in the m/z 50-1000 range. Hystar (Bruker) software was used for system control and data acquisition.

Data pre-processing

The data files generated after LC-HRMS analysis were converted to more exchangeable format NetCDF files (.cdf) using a conversion function from the Data Analysis software program (Bruker). The converted data were exported in the open-source XCMS software with the following parameters: *fwhm, step, steps, mzdiff, mzwid* and *minfrac* that were set at 10, 0.1, 5, 0.1, 0.1 and 0.7, respectively. Feature extraction was performed with matchfilter.

To control the global extraction of features by XCMS, Target Analysis software (Bruker) was used.

The first data set, obtained in the positive ion ESI mode, contained 2229 extracted features, and the second, obtained in the negative ion ESI mode, contained 751 features. The filter step was based on a coefficient of correlation greater than absolute value 0.5 as well as an intensity ratio of the biological samples/blank greater than 5 for the first data set and greater than 3 for the second.

Statistics

PCA and PLSDA were performed using SIMCA P12+ (Umea, Sweden). Significant features were selected according to their respective VIP values > 1 calculated from the SIMCA software. PLSDA model validation was performed by a permutation test.

- *Participant Q5 /T1*

The Ultra performance liquid chromatography-Quadrupole time-of-flight mass spectrometer system (UPLC-QTof Synapt-2 MS) (Waters Corp., Milford, MA, USA) was used to carry out our metabolomic studies. Analysis of extracts was performed using the UPLC-QTof Synapt MS, and the data were recorded with MassLynx software (Waters Corp.). The samples were run in a randomised order using a HSS-T3C18 column (2.1 mm × 50 mm, 1.8 µm particle size, Waters Corp.). The chromatographic separations were performed at a flow rate of 0.4 mL/min.  The column was eluted with 0.1% acetic acid in water (A) and 0.1% acetic acid in acetonitrile (B).  Samples (5 µL) were injected onto the column at 100% A held for 1 min and subsequently ramped to 90% B over 10 min, then held for 2 min before a rapid return to 100% A and an equilibration for 2 min.  Centroided mass spectra were acquired in the m/z ratio of 80-1200 Th using the mass spectrometer by separate injection with positive and negative electrospray dual scan LCMS modes with a scan time of 0.3 sec per channel.  Data obtained in the MassLynx software format were converted to NetCDF files for further data analysis using a proprietary conversion program (DataBridge). Further details of the data treatment are given in the next section.

HPLC-ToF analysis was conducted using a Phenomenex Kinetex C18 2.6 µm 100 mm x 2 mm column (Macclesfield, UK) coupled to a Bruker MicroTOF mass spectrometer in electrospray positive and negative ionisation modes (Coventry, UK). Chromatographic separations were performed at a flow rate of 0.25 mL/min, and the column was eluted with 0.1% formic acid in water (A) and 0.1% formic acid in acetonitrile (B).  Samples (5 µl) were injected onto the column at 98% A and maintained at 98% A for 2 min, then subsequently ramped to 80% B over 8 min, followed by a ramp to 98% B over 14 min, and held at 98% B for 1 min before a rapid return to 98% A and an equilibration for 6 min. The column temperature was maintained at 30°C, and the temperature of the samples was maintained at 4°C.  Centroided mass spectra were acquired in the m/z 80-1100 Th using the mass spectrometer in positive and negative electrospray scanning modes (run separately). The optimised settings comprised a capillary voltage at 4200 V, a capillary exit at 90 V and a hexapole RF at 90 V. Accurate mass calibration was achieved by the measurement of sodium formate at the start and end of each chromatographic run.  The samples were analysed in a randomised order, with the pooled sample analysed 5 times at the start of the sequence, followed by the metabolite, the blank and then the samples, with the pooled sample repeated every 6 samples.

For both the TOF and QTOF analyses, the raw LC/MS dataM_T_ values were analysed using the XCMS programme (<http://masspec.scripps.edu/xcms/xcms.php>). The basic output of the XCMS programme consists of a Table of intensities for the detected peaks in each sample, with the peaks labelled by the M_T_ value (where M is the m/z value and T is the retention time in sec). The program also carries out a t-test for significant differences between the samples from the two nominated groups and ranks the M_T_ values in order of the t-statistic (or p-value). The Table of intensities provided may also be used outside the XCMS program for a range of further statistical analyses, including both univariate methods (taking one M_T_ variable at a time) and multivariate methods (taking all variables simultaneously). The XCMS conditions used to deconvolute the raw LCMS data were the default ones, with the matchfilter algorithm used for feature detection, except for the group bandwidth (bw=10 for TOF and bw=5 for QTOF).

*Statistics*

Statistical analyses were performed using in-house Matlab® code. PCA was first performed to detect any group separation based on NMR signal variability. This method also enabled the detection of any excluded outliers, defined as observations situated outside the 95% confidence region of the model.

An orthogonal projection to latent-structure (OPLS) analysis was run to discriminate the two groups. Variables with VIP > 1.5 were considered as discriminant.

- *Participant Q6/O2*

For Test #1, the LC-MS measurements were performed on an Ultimate 3000 U-LC system (Dionex) coupled to a TLQ Orbitrap XL instrument (Thermo Fisher Scientific) equipped with an electrospray ion source (ESI).

Samples were loaded on a Waters XTerra MS C18 (2.1 x 5 mm, 3.5 µm) pre-column and separated on a Waters XTerra MS C18 (1.0 x 150 mm, 3.5 μm) column. The HPLC pump was operated at a flow rate of 40 μl/min. The eluents used were (A) 0.1% formic acid in Milli-Q water, (B) 0.1% formic acid in acetonitrile and (C) 0.1% formic acid in methanol. The linear gradient used to achieve analyte separation was as follows: 0-1.5 min at 95% A, 5% C; 10 min at 2% A, 48% B, 50% C; 10-11 min at 2% A, 48% B, 50% C; 11.1 min at 2% A, 98% B; 11.1-12 min at 2% A, 98% B; 12 min at 2%A, 98%B; 12.1 min at 98%A, 2%C; and 12.1-17 min at 98% A, 2% C.

The operating conditions of the LTQ Orbitrap XL mass spectrometer were as follows: capillary voltage, 45 V; tube lens voltage, 135 V; spray voltage, 3.5 kV (+ or – depending on the polarity); transfer capillary temperature, 200°C. The instrument was operated either in the positive or negative ESI mode. The FTMS (Orbitrap) analyser was calibrated using a solution of caffeine, MRFA (L-methionyl-arginyl-phenylalanyl-alanine acetate x water) and Ultramark 1621 in the range *m*/*z* 50-1000. LCMS acquisition was performed over the full *m*/*z* range with a scan time of 0.7 s. The resolution used was 30,000 FWHM and the mass accuracy was in the range of 1-5 ppm.

The LC-MS system was operated using XCalibur 2.0.7 software (for LCMS) and Chromeleon 6.8 software (for LC). LC-MS data were generated in the centroid mode. Two batches of analyses were considered (in ESI positive and ESI negative modes), and raw data files (*.RAW) were generated.

For Test #2, the LC-MS analyses were performed on an ultra performance liquid chromatography system (Acquity UPLC, Waters) coupled on-line with a hybrid quadrupole time-of-flight mass spectrometer (Q-TOF Premier, Waters) equipped with an electrospray ionisation source (ESI).

A 96-well plate containing samples was placed on the autosampler of the UPLC system. Five μl of sample was loaded on a Waters Acquity UPLC BEH C18 (1.0 x 100 mm, 1.7 μm) column. The binary UPLC pump was operated at a flow rate of 40 μl/min. The eluents used were (A) 0.1% formic acid in Milli-Q water and (B) 0.1% formic acid in acetonitrile. The linear gradient used to achieve analyte separation was as follows: 0-0.5 min at 100% A; 0.5-5 min at 100-70% A; 9-12 min at 70-0% A; 12-14 min at 0% A; and 14-16 min at 0-100% A. The column was re-equilibrated for 2 min with 100% A prior to the next injection.

The operating conditions of the Q-TOF mass spectrometer were as follows: capillary voltage, 3.5 kV (+); sample cone voltage, 38 V; extraction cone, 3.0 kV; source temperature, 80°C; desolvation temperature, 150°C; cone flow, 25 L/h; desolvation flow, 450 L/h. For the quadrupole and the collision cell, the parameters were as follows: LM resolution, 5.0; HM resolution, 15.0; ion energy, 1.5 V; cell entrance, 2.0 V; gas flow 0.10 mL/min. Time-of-flight (TOF) was performed in the continuous extraction mode. In the non-linear mode (W mode), an accelerating voltage of 9.0 kV and a reflectron voltage of 2.0 kV were used for the TOF. The microchannel plate (MCP) value of the detector was set to 650 V. The instrument was operated in the positive ESI mode. The TOF analyser was calibrated using a solution of sodium formate in the *m*/*z* range of 50-1000. Mass spectra (MS mode) were collected over the full *m*/*z* range with a scan time of 0.5 s and an interscan time of 0.05 s. The resolution used was 17,000 FWHM (*i.e.*, an m/z 500 ion peak has a width at half height of m/z 0.03) in the W mode. The lockspray device gave a high mass accuracy for internal calibration (5 ppm).

The LC-MS system was operated using MassLynx 4.1 software (Waters), and the LC-MS data were generated in the continuum mode. Raw data files (*.RAW) were generated.

Data storage and handling

Each of the two analysis batches (in the ESI positive and negative modes) was associated with a *.CSV file that contained the following information on the standardised analysis sequence: file name (unique name in a sequence), sample code (unique code attributed to a sample) and sample name (standard, blank or sample). Raw LC-MS data were converted into *.CDF files using Sync Toy 2.1 (Microsoft). The *.CDF files were placed on a server and were ready for data pre-processing.

Data pre-processing

LC-MS data were pre-processed with the "XCMS" package of R, which was implemented in-house. The software extracted and nonlinearly aligned the retention times and the accurate masses of the LC-MS produced peaks in the time range of 2-15 min. The inputs comprised three dimensional raw datasets (m/z, rt, intensity), and the deliverables were peak intensity tables (features, statistical parameters).

The parameters of the extraction method "matchedFilter" were: rtcut = c(2,15)*60, profmethod = "bin", fwhm = 30, max = 10, snthresh = 6, step = 0.01, steps = 2, mzdiff = 0.025.

For the grouping of the found peaks (method "density"), the used settings were: mzppm = 10, mzabs = 0, minsamp = 1, minfrac = 0.5, bw = 5, minfrac = 0.7, minsamp = 2, mzwid = 0.013, mzVsRTbalance = 10, mzCheck = 0.2, rtCheck = 15, kNN = 10.

For the function "Groupval", parameters were as follows: GMeth = c("medret","maxint"), GVal = "into", GInt = "into", DiffClass = c("AllBinaries","ANOVA1w","ANOVANw"), KEGGComplete = 0, UseDiff = c(p = 0.01, fc = 5, camera = 0.05)).

For retention time correction, the method "obiwarp" was used. The parameters were:

GroupBW = 1, NumPass = 1, missing = 1, smooth = "loess", extra = 1, span = 0.2, profStep = 1, response = 1, factorDiag = 2, factorGap = 1, localAlignment = 0, initPenalty = 0.

Normalisation of the signal was performed using the method "MSTUS". The parameters were: pSam = 80, quant = 50, Trim = 0.125.

The CAMERA package was used, and the rules were set either for the positive or the negative ESI mode.

A peak intensity Table was generated for each of the two datasets (ESI positive and negative) and used for data analysis (statistical treatment).

*Statistics*

SIMCA P (Umea, Sweden) was used to run PCA and PLSDA analyses. Permutation tests enabled model validation. Features were selected according to their VIP values (>1) and fold change (>2).

- *Participant O1*

**Test 1:**

## Liquid chromatography and mass spectrometry

Analyses were performed using a Nexera liquid chromatographic system (Shimadzu, Marne-la-Vallée, France) coupled to an Exactive mass spectrometer (Thermo Fisher Scientific, Courtaboeuf, France) fitted with an electrospray source operated in the positive ion mode. The software interface was Xcalibur (version 2.1.) (Thermo Fisher Scientific, Courtaboeuf, France). The ultra high performance liquid chromatographic (UHPLC) separation was performed on a Hypersil GOLD C_18_ 1.9 μm, 2.1 mm × 150 mm column (Thermo Fisher Scientific, Courtaboeuf, France) equipped with an online prefilter (Interchim, Montlucon, France). The mobile phases were (A) 100% water and (B) 100% acetonitrile containing 0.1% formic acid. After an isocratic step of 2 min at 100% phase A, a linear gradient from 0% to 100% B was run over the next 11 min with a mobile phase flow of 500 μl/min. Returning to 100% A at 15.50 min, the column was then allowed to equilibrate for 3.5 min, leading to a total run time of 19 min.

Mass spectra were recorded from 65 Th up to 1200 Th in the positive ion mode. In the positive ion mode, the electrospray voltage was set to 4.8 kV, the capillary voltage was set to 25 V, and the tube lens was offset to 90 V. The sheath and auxiliary gas flows (both nitrogen) were optimised at 60 and 20 arbitrary units (a.u.), respectively, and the drying gas temperature was set to 275°C. The mass spectrometer was calibrated before each analysis using a calibration solution provided by the manufacturer (external calibration). For singly charged ions, the mass resolution power of the analyser was set to 50,000 (m/Δm, FWHM at 100 Th) and the mass accuracy was within a 5 ppm range.

## Data processing

All raw data were manually inspected using the Qualbrowser module of Xcalibur version 2.0.7 (Thermo Fisher Scientific, Courtaboeuf, France), and its chemical formula generator was used to provide elemental compositions.

Automatic peak detection and integration were performed using the XCMS software package^26^ (version 1.14.1 running under R version 2.8.1). Raw files were first converted to netCDF format with Xconvert (Thermo Scientific, Courtaboeuf, France). The R language was installed on a Dell Eight-core Intel Xeon 3.00 GHz Processor with 16 Go RAM running Linux (Centos 5.2 x86_64). The matchedFilter algorithm was used, and default values were set for all parameters except for “fwhm”, “step”, “steps”, “mzdiff”, “mzwid”, and “minfrac,” which were set, respectively, at 4, 0.01, 2, 0.01, 0.01 and 0.7 for both group functions.

Public database (KEGG, HMDB and Metlin) annotations were performed on datasets resulting from the XCMS process with a home-made informatics tool developed in R language. Annotations were carried out by matching the measured accurate masses ± 10 ppm with the theoretical ones of public databases. Data were also annotated using an in-house spectral database.

Statistical analyses

The resulting data from the XCMS process were first mean-centred and scaled either to unit variance or to Pareto variance, then introduced into SIMCA-P11 (Umetrics, Umea, Sweden) for multivariate analyses using principal component analysis (PCA) and projection to latent structure discriminant analysis (PLS-DA). This software returns a variable importance on projection (VIP) score, which reflects the contribution of the variables to the model. A variable was considered as important for the model when its VIP was above 1.0. The PLS-DA models were validated using the cross-validation function of SIMCA-P11 and by permutation tests (k = 100).

**Test 2:**

A quality control sample consisting of a pool of plasma samples analysed in the course of this study was injected every 10 samples to check the performance of the analytical system in terms of retention times, accurate mass measurements, and signal intensities. This QC sample was also subsequently diluted 2-fold, 4-fold, and 8-fold and analysed in triplicate.

Liquid chromatography and mass spectrometry

An Accela LC system (ThermoFisher Scientifics, Courtaboeuf, France) was coupled to an LTQ-Orbitrap instrument for sample introduction and separation. The UHPLC chromatographic separation was performed on a Hypersil GOLD C_8_ 1.9 μm, 2.1 mm × 150 mm column (Thermo Fisher Scientific, Les Ulis, France) equipped with an online prefilter (Interchim, Montlucon, France). The mobile phases were (A) 100% water and (B) 100% acetonitrile containing 0.1% formic acid. After an isocratic step of 2 min at 5% phase B, a linear gradient from 5% to 100% B was run over the next 11 min with a mobile phase flow of 500 μl/min. These proportions were kept constant for 12.5 min before returning to 5% B for 4.5 min.

Mass spectra were recorded from 75 µ up to 2000 µ in positive and negative ion mode. In the positive ion mode, the electrospray voltage was set to 5 kV, the capillary voltage was set to 15 V, and the tube lens was offset to 50 V. The sheath and auxiliary gas flows (both nitrogen) were optimised at 60 and 10 arbitrary units (a.u.), respectively, and the drying gas temperature was set to 275°C. In the negative ion mode, the electrospray voltage was set to -3 kV, and the capillary voltage and tube lens offset were set to -10 and -100 V, respectively; the sheath and auxiliary gas flows (both nitrogen) were 60 and 10 au, respectively, and the drying gas temperature was 275°C. For singly charged ions, the mass resolution power of the analyser was set to 30000 (m/Δm, FWHM at 400 Th), and the mass accuracy was within a 5 ppm range.

## Data processing

Please refer to test 1, except for XCMS parameters: “fwhm”, “step”, “steps”, “mzdiff”, “mzwid”, and “minfrac” which were set, respectively, at 4, 0.05, 2, 0.01, 0.01 and 0.4 for both group functions.

Statistical analyses

Please refer to test 1.

- *Participant O3*

Sample fingerprinting was performed on an Agilent 1200 HPLC system including an autosampler and a binary pump and coupled to a Finnigan LTQ-Orbitrap^TM^ hybrid mass spectrometer (Thermo Fisher Scientific, Bremen, Germany). Chromatographic separation was performed on a Hypersil-Gold column (100 mm x 2.1 mm x 1.9 µm particle size, Thermo Fisher Scientific). The mobile phase consisted of water containing 0.1% acetic acid (A) and acetonitrile containing 0.1% acetic acid (B). The used elution gradient (A:B, v/v) was as follows: 95:5 from 0 to 2.4 min; 75:25 at 4.5 min; 30:70 at 11 min; and 0:100 at 14 min. The injected volume was 5 µL, the flow rate was 0.4 mL/min and the temperature of the column was maintained at 37°C. The HPLC column was connected without splitting to the electrospray interface operating in positive or negative ion modes. In the positive ion mode, the electrospray voltage was set to 4.5 kV, the capillary voltage was set to 30 V, and the tube lens was offset to 100 V. The sheath and auxiliary gas flows (both nitrogen) were set to 55 and 6 arbitrary units (a.u.), respectively, and the drying gas temperature was set to 325°C. In the negative ion mode, the electrospray voltage was set to -4 kV, and the capillary voltage and tube lens offset were set to -20 and -90 V, respectively. The gas flows were identical to those used in the positive ion mode. Mass spectra were recorded from 65 Th to 1000 Th at a resolution of 30000 (FWHM at m/z 400). Mass spectra were acquired in the centroid mode.

Data processing

Following their acquisition by a given analytical tool, the metabolomic fingerprints were deconvoluted to allow the conversion of the three-dimensional raw data (m/z, retention time, ion current) to time- and mass-aligned chromatographic peaks with associated peak areas. Xcalibur^®^ software (Thermo Fisher Scientific) was used to convert the original Xcalibur data files (*.raw) to a more exchangeable format (*.cdf). Data processing was then performed using the open-source XCMS software. XCMS parameters for the R language were implemented in an automated script. MatchFilter was used for the peak picking. The interval of the m/z value was set to 0.1, the signal to noise ratio threshold was set to 6, the group band-width was set to 15 and the minimum fraction was set to 0.75.

Statistics

Data were log transformed, Pareto-scaled and analysed by PCA and PLSDA using SIMCA P+12 (Umea, Sweden). Models were validated using cross validation and permutation tests. Significant features were retained using VIP values scores calculated by the SIMCA algorithm over 1.

- *Participant O4*

Sample fingerprinting was performed on a Dionex Ultimate 3000 RSLC system including an autosampler and a tertiary pump and coupled to a Finnigan LTQ-Orbitrap^TM^ hybrid mass spectrometer (Thermo Fisher Scientific, Bremen, Germany). Chromatographic separation was performed on an Acclaim 120 C18 column (100 mm x 2.1 mm x 2.2 µm particle size, Dionex). The mobile phase consisted of water containing 0.1% acetic acid (A) and acetonitrile containing 0.1% acetic acid (B). The used elution gradient (A:B, v/v) was as follows: 80:20 from 0 to 5 min; 95:5 at 15 min and hold for 10 min; 80:20 at 26 min and hold for 4 min. The injected volume was 5 µL, the flow rate was 0.25 mL/min, and the temperature of the column was maintained at 20°C. The UHPLC column was connected without splitting to the electrospray interface operating in positive ion mode. In the positive ion mode, the electrospray voltage was set to 3.5 kV, the capillary voltage was set to 45 V, and the tube lens was offset to 130 V. The sheath and auxiliary gas flows (both nitrogen) were set to 5 arbitrary units (a.u.), and the drying gas temperature was set to 300°C. Mass spectra were recorded from 50 m/z to 1000 m/z at a resolution of 30,000 (FWHM at m/z 400). Mass spectra were acquired in the centroid mode.

*Data processing*

Following their acquisition by Xcalibur^®^ software (Thermo Fisher Scientific), the metabolomic fingerprints were deconvoluted to allow the conversion of the three-dimensional raw data (m/z, retention time, ion current) to time- and mass-aligned chromatographic peaks with associated peak areas. Massmatrix File Conversion was used to convert the original Xcalibur data files (*.raw) to a more exchangeable format (*.mzXML). Data processing was then performed using the open-source XCMS software. XCMS parameters for the R language were implemented in an automated script. CentWave was used for the peak picking. The interval of the m/z value was set to 0.1, the signal to noise ratio threshold was set to 10, the group band-width was set to 10 and the minimum fraction was set to 0.75.

Identification of the different metabolites was carried out using the Metlin database.

**Online Resource Figure 1.** flow chart of the statistical design of the experiment

The first part of the experiment was to generate data from the various partners (**panel A**); the second part was dedicated to the data analysis, with the main focus put on the comparison among the metabolic profiling (**panel B**).

**Online resource Figure 2. Comparison of the observed vs modified RV coefficients.**

Comparison of the Average observed RV coefficients (*Lavit, C.; Escoufier, Y.; Sabatier, R.; Traissac, P. Computational Statistics & Data Analysis 1994, 18, 97-119***)** to the modified-RV coefficients (grey) (*Smilde, A. K.; et al. v. Bioinformatics* ***2009****, 25, 401-405*) of each instruments with all others (plot symbols), and distribution of average RV coefficients based on 100 random samplings for each dataset (box plots) (i.e. features extracted by instrument). **Panel A**, Test#1, high biological contrast, **panel B**, Test#2, low biological contrast. Open squares, NMR (N), circles, Orbitraps (O) operating in positive (P) or negative (N) mode, squares, QTOF (Q) operating in positive (P,) or negative (N) mode, triangle, TOF (T) operating in positive (P) or negative (N) mode.


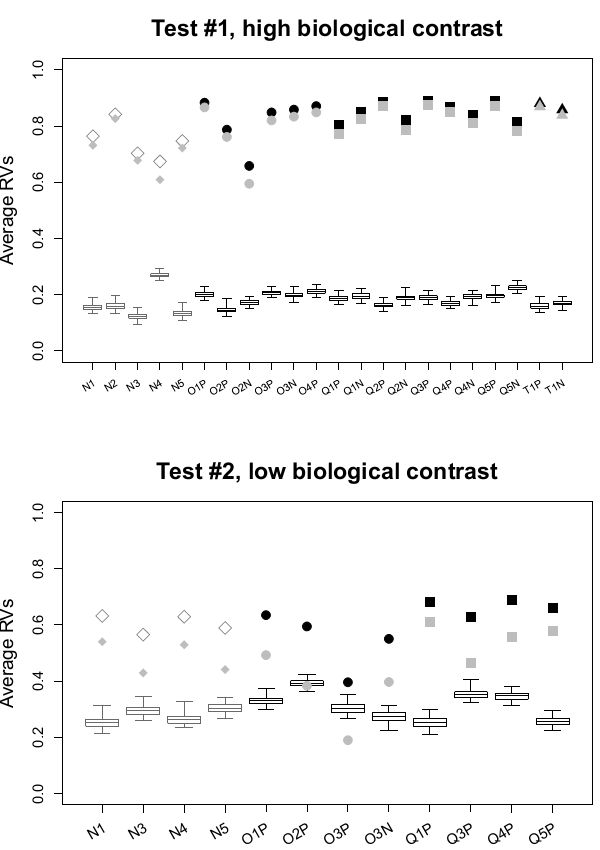


**Online resource Figure 3. Results after NMR2 was normalized on total spectral intensity.**

**A,**  Average RV coefficients (based on observed data) of each instruments with all others; **B**, Individual Common Component and Specific Weights Analysis scores (higher panels) and saliences -or loadings- (lower panels) calculated from Test #1 datasets. Symbol keys: open diamonds denote NMR, black circles denote Orbitrap, squares denote QTOF, and triangles denote TOF; **C**, Correlations networks calculated from test#1 pair-wise RV coefficients matrix. Node labelling: N, NMR platforms, Q , QTOF mass spectrometer, O, Orbitrap mass spectrometer, T, TOF mass spectrometer. The P or N appended to the mass spectrometer identifier number denotes positive or negative ionization mode, respectively. Node shapes: hexagone for nuclear magnetic resonance platforms, ellipse for mass spectrometers. Node size is proportional to the number of features retained by each instrument. Node color: from green to red indicates an increasing node degree (number of edges per node). The edges represent RV coefficient values, with cut off values ≥0.791.

**A**


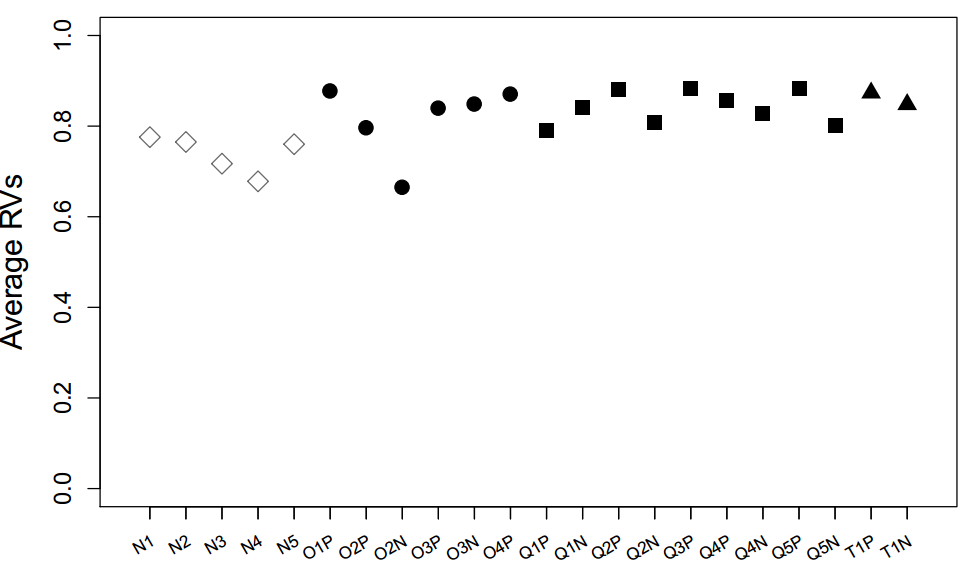


**B**


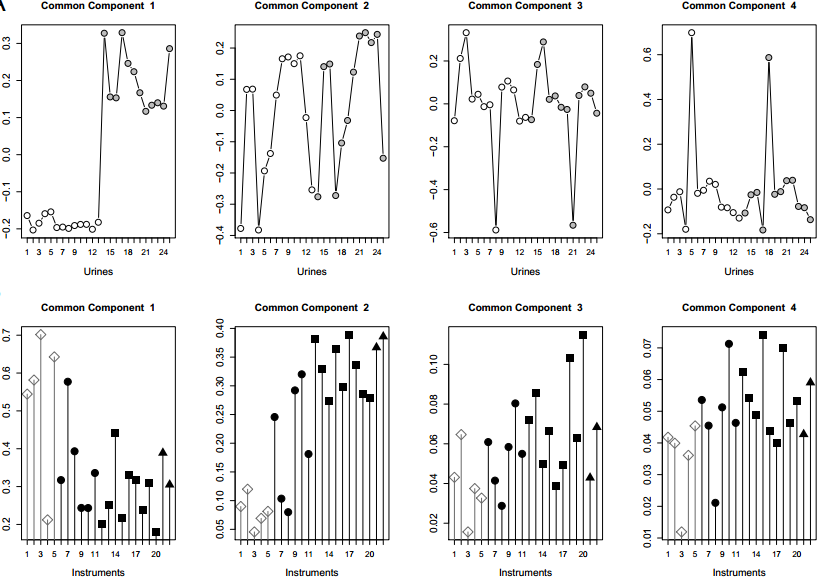


**C**


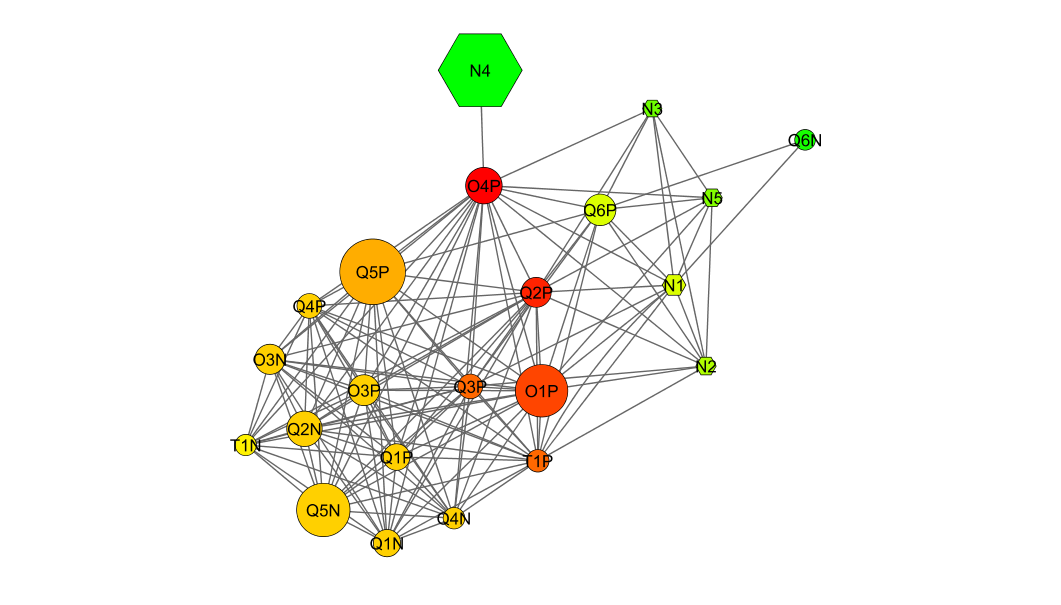


**Online resource Figure 4. Results after normalization of the non-spiked urines samples to the respective dilution factor.** Individual Common Component and Specific Weights Analysis scores (panel A) and saliences -or loadings- (panel B) calculated from Test #1 datasets. Symbol keys: open diamonds denote NMR, black circles denote Orbitrap, squares denote QTOF, and triangles denote TOF;


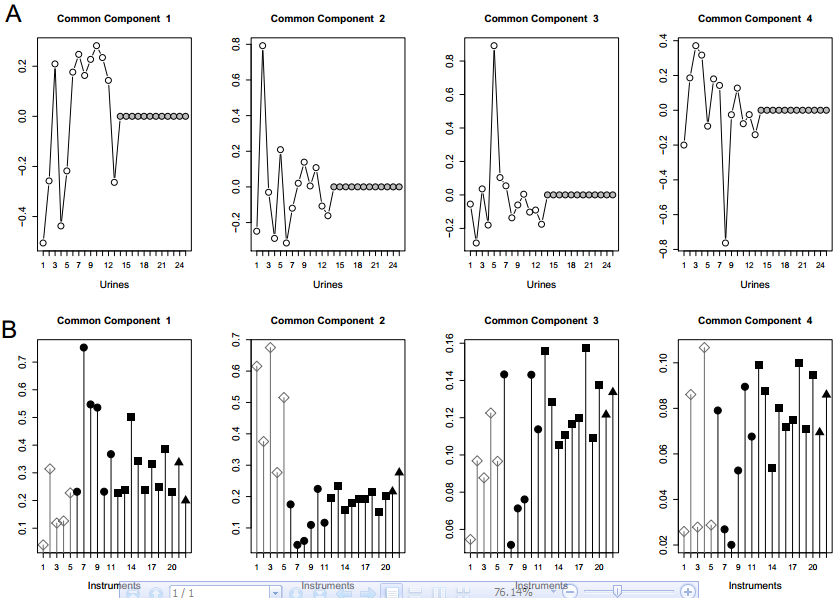


**Online resource Figure 5** Global and local scores calculated from the common component specific weight analysis of Test#1 dataset **(panel A**) and Test#2 dataset (**panel B**). Each sample is represented in the common space as the barycentre of each platform result (global CCSWA score), and the edges beaming out as the individual platform dispersion around this barycentre (specific or local CCSWA scores). The smaller the length of edges is, the more the samples are identical across all instruments. In Test #1, all instruments were able to detect the inter-individual variability (score 2) as well as the inter-group variability (score 1). In Test #2, the discrimination between control rats and vitamin D challenged rats was more tenuous. The second common dimension slightly opposed blood samples of the supplemented group (at the top) to those of the control group (at the bottom). Consequently, the common information extracted by all instruments represented mainly the inter-individual variability.


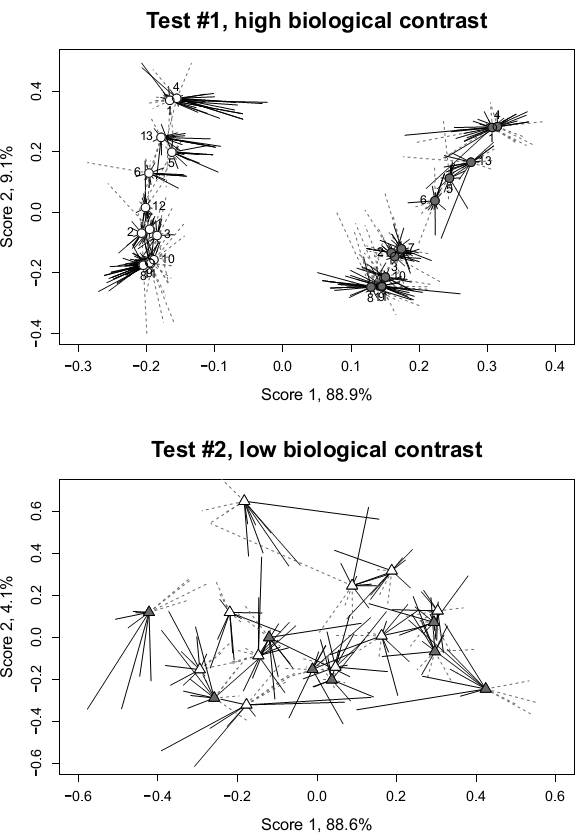


**A**

**B**

**Online resource Tables**

**Can We Trust Untargeted Metabolomics: Results of the Metabo-Ring initiative, a large-scale multi-instruments inter-laboratory study**

**Table 1.** Composition and final content of the standards mixture added to the urine samples

| **compounds** | **ChEBI identifier** | **occured in urine** | **concentration** | **spiked value for LCMS** | **spiked value for NMR** | **spiked value** | **spiked value** |
| --- | --- | --- | --- | --- | --- | --- | --- |
|  |  | **(HMDB)** | **(in mmol creatinine)** | **µmol/mmol creatinine** | **µmol/mmol creatinine** | **for LCMS (mM)** | **for NMR (mM)** |
| Ascorbic acid | CHEBI:29073 | yes | 0.5 ± 0.06 | 11.4 | 113.64 | 0.14 | 1.42 |
| Citrulline | CHEBI:18211 | yes | 1.0 ± 0.7 | 11.4 | 114.3 | 0.14 | 1.43 |
| **Creatinine** | CHEBI:16737 | **yes** | **12.5 ± 8 mM** | 17.7 | 177,0 | 0.22 | 2.21 |
| Taurine | CHEBI:15891 | yes | 36 ± 30 | 16.0 | 160.0 | 0.2 | 2.0 |
| Uric acid | CHEBI:27226 | yes | 200 ± 80 | 11.9 | 119,0 | 0.15 | 1.49 |
| Caffeine | CHEBI:27732 | yes | 1 | 10.3 | 103.1 | 0.13 | 1.29 |
| L-Glutaric acid | CHEBI:17859 | yes | 2 | 15.2 | 151.5 | 0.19 | 1.89 |
| Inosine | CHEBI:17596 | yes | 1 ± 0.6 | 7.5 | 74.6 | 0.09 | 0.93 |
| L-Isoleucine | CHEBI:17191 | yes | 3 to 17 | 15.3 | 152.7 | 0.19 | 1.91 |
| L-Leucine | CHEBI:15603 | yes | 6 to 19 | 15.3 | 152.7 | 0.19 | 1.91 |
| Pyroglutamic acid | CHEBI:18183 | yes | 14 ± 7 | 15.5 | 155,0 | 0.19 | 1.94 |
| L-Methionine | CHEBI:16643 | yes | 1.7 ± 1 | 13.4 | 134.2 | 0.17 | 1.68 |
| Methylmalonic acid | CHEBI:30860 | yes | 3.5 ± 2.6 | 16.9 | 169.5 | 0.21 | 2.12 |
| N-methylhistidine | CHEBI:50601 | yes | 5 to 70 | 11.8 | 118.3 | 0.15 | 1.48 |
| Aminobenzoic acid | CHEBI:22495 | yes | 0.15 to 0.6 | 14.6 | 146,0 | 0.18 | 1.82 |
| L-Phenylalanine | CHEBI:22495 | yes | 4.5 ± 1.9 | 12.1 | 121.2 | 0.15 | 1.52 |
| L-Proline | CHEBI:17203 | yes | 3 ± 1.9 | 17.4 | 173.9 | 0.22 | 2.17 |
| Riboflavin | CHEBI:17015 | yes | 0.2 ± 0.2 | 5.3 | 53.2 | 0.07 | 0.66 |
| Adenosine | CHEBI:16335 | yes | 4.7 ± 7 | 7.5 | 74.9 | 0.09 | 0.94 |
| Adenine | CHEBI:16708 | yes | 0.6 to 5.1 | 14.8 | 148.1 | 0.19 | 1.85 |
| Adipic acid | CHEBI:30832 | yes | 1.6 to 14 | 13.7 | 137,0 | 0.17 | 1.71 |
| Azelaic acid | CHEBI:48131 | yes | 2.2 ± 1.7 | 10.6 | 106.4 | 0.13 | 1.33 |
| Caffeic acid | CHEBI:17395 | yes | 0.13 ± 0.032 | 11.1 | 111.1 | 0.14 | 1.39 |
| L-Tryptophan | CHEBI:16828 | yes | 13.5 ± 7.4 | 9.8 | 98,0 | 0.12 | 1.23 |
| L-Tyrosine | CHEBI:17895 | yes | 7.4 ± 2.3 | 11,0 | 110.5 | 0.14 | 1.38 |
| Uracil | CHEBI:17568 | yes | 12.3 ± 5.8 | 17.9 | 178.6 | 0.22 | 2.23 |
| Uridine | CHEBI:16704 | yes | 0.2 ± 0.1 | 8.2 | 82,0 | 0.1 | 1.02 |
| Chenodeoxycholic | CHEBI:16755 | no |  | 5.1 | 50.9 | 0.06 | 0.64 |
| Cholic acid | CHEBI:16359 | yes | 2 to 7 | 4.9 | 48.9 | 0.06 | 0.61 |
| Cortisone | CHEBI:16962 | yes | 0.01 ± 0.001 | 5.6 | 55.6 | 0.07 | 0.69 |
| Deoxycholic acid | CHEBI:28834 | yes | 0.07 to 0.9 | 5.1 | 50.9 | 0.06 | 0.64 |
| Glycocholic acid | CHEBI:17687 | yes | 0.4 ± 0.4 | 4.3 | 42.9 | 0.05 | 0.54 |

**Table 2.** Nutrients composition of the based-diet given to the rats (test#2).

| **Food component** | **amount** |  |
| --- | --- | --- |
| Casein | 20.5% |  |
| Saccharose | 36.8% |  |
| Lard | 35% |  |
| Mineral mix 205B | 5.9% |  |
| Vitamine mix 200 | 1% |  |
|  |  |  |
| **Nutritional composition** |  |  |
| Proteins | 17.4% | 697kcal/kg |
| Lipids | 35.8% | 3222kcal/kg |
| Carbohydrates | 35% | 1398kcal/kg |
|  |  |  |
| **Mineral composition** | mg/kg |  |
| Calcium | 6350 |  |
| Phosphore | 4600 |  |
| Sodium | 2300 |  |
| Chloride | 6600 |  |
| Potassium | 3150 |  |
| Magnesium | 850 |  |
| Sulfite | 110 |  |
| Iron | 86 |  |
| Copper | 73 |  |
| Manganese | 460 |  |
| Zinc | 260 |  |
| Iodine | 3.6 |  |
| Cobalt | 0.6 |  |
|  |  |  |
| **Vitamin composition** |  |  |
| Vitamin A | 20000UI/kg |  |
| Vitamin E | 175mg/kg |  |
| Vitamin K3 | 17mg/kg |  |
| VitaminB1 | 20mg/kg |  |
| VitaminB2 | 15mg/kg |  |
| Vitamin PP | 100 mg/kg |  |
| Vitamin B5 | 7 mg/kg |  |
| Vitamin B6 | 10 mg/kg |  |
| Vitamin B9 | 5 mg/kg |  |
| Vitamin H | 0.3 mg/kg |  |
| Vitamin B12 | 0.05 mg/kg |  |
| Vitamin C | 0.8 mg/kg |  |
| Choline | 1360 mg/kg |  |

**Table 3.** Standard molecules added to the urine samples and identified by each partner.

| **compounds** | **NMR** | | | | **ESI +ve** | | | | | | | | | **ESI -ve** | | | | | |
| --- | --- | --- | --- | --- | --- | --- | --- | --- | --- | --- | --- | --- | --- | --- | --- | --- | --- | --- | --- |
|  | **N1** | **N2** | **N3** | **N5** | **Q1** | **Q3** | **Q4** | **Q5** | **Q6** | **O1** | **O3** | **O4** | **T1** | **Q1** | **Q4** | **Q5** | **Q6** | **O3** | **T1** |
| ascorbic acid |  | X |  |  |  |  |  |  |  |  |  |  |  |  |  |  |  |  |  |
| citrulline | X | X | X | X | X |  | X |  |  | X |  |  |  |  |  |  |  |  |  |
| creatinine | X | X |  | X |  |  |  |  |  | X | X |  |  |  |  |  |  | X |  |
| taurine | X | X |  | X |  |  |  |  |  |  | X |  |  |  |  | X | X | X |  |
| uric acid |  |  |  |  |  | X |  | X |  |  |  |  | X |  |  |  | X | X |  |
| caffeine | X | X | X |  | X | X | X | X | X | X | X |  |  |  |  |  |  |  |  |
| glutaric acid | X | X | X | X |  |  |  |  | X | X |  |  | X |  | X |  | X | X | X |
| inosine | X | X | X | X | X |  | X | X | X | X | X |  | X | X | X | X |  | X | X |
| isoleucine | X | X | X | X | X |  | X | X | X | X | X |  |  |  |  | X | X | X |  |
| leucine | X | X | X | X | X |  | X | X | X | X | X |  | X |  |  | X | X | X |  |
| pyroglutamic acid | X | X |  | X |  |  | X |  |  | X | X |  | X |  |  |  | X | X | X |
| methionine | X | X |  | X | X | X | X | X | X | X | X |  | X |  |  |  |  |  |  |
| methylmalonic acid | X | X | X | X | X |  |  | X |  |  |  | X | X | X |  | X |  | X | X |
| N-methylhistidine |  |  |  |  |  | X | X |  |  | X | X |  | X |  | X | X |  | X |  |
| aminobenzoic acid | X | X | X | X | X | X | X | X | X | X | X |  | X |  |  |  |  | X |  |
| phenylalanine | X | X |  | X | X |  | X | X | X | X | X | X | X | X | X | X |  | X | X |
| proline | X | X | X | X |  |  | X | X |  | X | X |  | X |  |  | X |  | X |  |
| riboflavin |  |  |  | X | X | X | X | X | X | X | X | X | X | X | X | X |  | X | X |
| adenosine | X | X |  |  | X | X | X | X | X | X | X | X | X |  | X | X |  | X | X |
| adenine | X | X |  | X | X | X | X | X | X | X | X |  | X | X |  | X | X | X |  |
| adipic acid | X |  |  | X |  |  | X | X | X | X | X | X | X | X | X | X |  | X | X |
| azelaic acid | X | X | X | X | X | X | X |  | X | X |  | X | X | X |  | X | X | X | X |
| caffeic acid |  | X | X | X | X |  |  | X |  |  |  |  | X |  |  | X |  | X | X |
| tryptophan |  | X | X | X | X | X | X | X | X | X | X | X | X | X | X | X | X | X |  |
| tyrosine | X | X | X | X | X | X | X | X | X | X | X |  | X |  | X |  |  | X |  |
| uracil |  | X |  |  |  |  | X | X | X |  |  |  |  |  |  | X |  |  |  |
| uridine | X | X | X | X |  |  | X |  | X | X | X |  |  | X | X | X | X | X |  |
| chenodeoxycholic acid |  |  |  |  | X |  |  |  | X |  | X | X |  |  | X | X | X | X | X |
| cholic acid | X |  |  | X | X |  | X |  | X | X | X | X | X | X | X | X | X | X | X |
| cortisone |  |  | X |  | X | X | X | X | X | X | X | X | X | X | X | X |  |  | X |
| deoxycholic acid |  |  | X | X | X |  |  |  | X | X |  |  |  | X | X | X | X |  |  |
| glycocholic acid |  |  |  |  | X | X | X | X | X | X | X | X | X | X | X | X | X | X | X |
| Total Number of ticked compounds | 22 | 24 | 16 | 23 | 21 | 13 | 23 | 20 | 22 | 25 | 23 | 11 | 22 | 13 | 15 | 22 | 14 | 25 | 14 |

**Table 4.** Putative metabolites annotation of spectral features discriminating individual#5. For mass spectrometers, ionisation mode are either positive, POS, or negative, NEG. The features are displayed either as an integer of the m/z value (M) and retention time (T) for mass spectrometers, or as a 1 [H] chemical shift for NMR. N*i* stands for *NMRi*, Q*i* for QTOF*i*, Oi for orbitrap *i*.

| **Platform ID** | **ionization mode** | **Features ID** | **Adducts type** | **Generated**  **formula** | **Annotated formula** | **Metabolites ID proposition** |
| --- | --- | --- | --- | --- | --- | --- |
| Q1 | POS | M533T564 | [2M+H]1+ | C36H37O4 | C18H18O2 | 3-hydroxy-estra-1,3,5(10),6,8-pentaen-17-one |
| Q1 | POS | M535T525 | [M+Na2-H]+ | C27H44NaO9 | C21H35O2 | Epipregnanolone glucuronide |
| Q1 | POS | M533T585 | [M+Na]1+ | C27H42NaO9 | C21H34O3 | 21-hydroxyallopregnanolone-glucuronide |
| Q1 | POS | M528T601 | [M+NH4]1+ | C27H46NO9 | C21H34O3 | Dihydroxy-pregnane-beta-one-glucuronide |
| Q1 | POS | M528T554 | [M+NH4]1+ | C27H46NO9 | C21H33O2 | Dihydroxy-pregnane-beta-one-glucuronide |
| Q1 | POS | M513T626 | [M+H]1+ | C27H45O9 | C21H36O3 | 5beta-Pregnane-3alpha,17alpha,20alpha-triol |
| Q1 | POS | M204T623 | [M+H -H20]1+ | C8H16NO6 | C8H15NO6 | N-Acetyl-D-glucosamine (glucuronide like fragment) |
| Q1 | POS | M549T486 | [M+H]1+ | C21H35O4 | [C21H34O4](http://www.lipidmaps.org/tools/ms/iso2d_Ag.php?formula=C21H34O4) | 9-deoxy-9-methylene-PGE2 glucuronide |
| Q1 | POS | M571T586 |  |  |  | unknown |
| Q1 | POS | M487T469 | [M+H -H20]1+ | C18H23O2 | C18H24O3 | hydroxyestradiol glucuronide |
| Q1 | POS | M467T330 | [M+Na]1+ |  |  | unknown |
| Q1 | POS | M506T349 | [M+NH4]1+ | |  | unknown |
| Q1 | POS | M531T567 | [M+H]1+ | C21H33O3 | C21H32O3 | Hydroxypregnenolone glucuronide |
| Q1 | POS | M204T546 | [M +H-H20]1+ | C8H14NO5 | C8H15NO6 | N acetyl glucosamine residue |
| Q1 | POS | M423T298 |  |  |  | unknown |
| Q1 | POS | M231T83 | [2M+Na]1+ | C8H16NaO6 | C4H8O3 | a-Hydroxyisobutyric acid |
| Q1 | POS | M285T666 | [M+Na]1+ | C24H32NaO5 | C24H32O5 | 7alpha-Hydroxy-3,12-dioxochola-1,4-dien-24-oic Acid |
| Q1 | POS | M626T586 |  |  |  | unknown sulfoconjugate |
| Q1 | POS | M381T33 | [M+Na]1+ | C12H22NaO11 | C12H22O11 | sucrose |
| Q1 | POS | M363T492 | [M+H]1+ | C21H31O5 | 21H30O5 | cortisol or aldosterone |
| Q1 | POS | M352T29 |  |  |  | unknown |
| Q1 | POS | M204T590 | [M+H-H20]1+ | C8H14NO5 | C_8_H_15_NO_6_ | N acetyl glucosamine residue |
| Q1 | POS | M533T602 | [M+H]1+ | C21H35O3 | C21H34O3 | 21-hydroxyallopregnanolone glucuronide |
| Q1 | POS | M349T277 | [M+Na]1+ |  |  | unknown |
| Q1 | POS | M305T171 | [M+Na]1+ |  |  | unknown |
| Q1 | POS | M533T592 | [M+H]1+ | C21H35O3 | C_21_H_34_O_3_ | Tetrahydrodeoxycorticosterone glucuronide |
| Q1 | NEG | M493T628 | [M-H]1- | C27H41O8 | C27H42O8 | Pregnanolone 3-β-D-Glucuronide |
| Q1 | NEG | M602T637 |  | C26H44N5O11 |  | unknown |
| Q1 | NEG | M494T629 | [M-H-H20]1- | C27H41O8 | C27H44O9 | Pregnanetriol 3α-O-β-D-Glucuronide |
| Q1 | NEG | M511T572 | [M-H]1- | C27H43O9 | C27H44O9 | (3β,14β)-14-Hydroxy-20-oxopregn-5-en-3-yl β-D-glucopyranoside |
| Q1 | NEG | M603T637 |  | C26H44N5O11 |  | unknown |
| Q1 | NEG | M463T477 | [M-H]1- | C9H17O2 | C9H16O2 | methyl octanoate glutathione conjugate |
| Q1 | NEG | M602T619 |  |  |  | unknown |
| Q1 | NEG | M495T621 | [M-H]1- | C27H43O8 | C27H44O8 | 3-alpha,20-alpha-dihydroxy-5-beta-pregnane 3-glucuronide |
| Q1 | NEG | M640T508 | [M-H]1- | C32H50NO12 | C32H51NO12 | (3a,5b,7a,12a)-24-[(carboxymethyl)amino]-1,12-dihydroxy |
|  |  |  |  |  |  | 24-oxocholan-3-yl-b-D-Glucopyranosiduronic acid |
| Q1 | NEG | M233T258 | [M+CH3COOH-1]1- | C9H5N4O2S | [C7H8O](http://maltese.dbs.aber.ac.uk:8888/hrmet/search/isotope.php?molform=C8H9O3&charge=1&nelec=1&rellim=1) | p-cresol sulfate |
| Q1 | NEG | M425T255 | [M+CH3COOH-1]1- | C22H33O8 | C20H30O6 | 20-Carboxyleukotriene B4 |
| Q1 | NEG | M400T630 |  |  |  | unknown |
| Q1 | NEG | M509T616 | [M-H]1- | C26H37N8OS |  | unknown |
| Q1 | NEG | M407T511 | [M-H +HCOOH]1- | C22H31O7 | [C21H30O5](http://maltese.dbs.aber.ac.uk:8888/hrmet/search/isotope.php?molform=C22H31O7&charge=1&nelec=1&rellim=1) | cortisol or aldosterone |
| Q1 | NEG | M222T29 | [M-H]1- | C5H4N9O2 |  | unknown |
| Q1 | NEG | M527T512 | [M-H]1- | C19H31N18O |  | unknown |
| Q6 | POS | M222T545 | [M+H]+ |  | C8H15NO6 | N-Acetyl-D-glucosamine |
| Q6 | POS | M335T570 | [M+H]+ |  | C21H34O3 | Tetrahydrodeoxycorticosterone or 3alpha,6alpha-Dihydroxy-5beta-pregnan-20-one or 21-hydroxyallopregnanolone or DIHYDROXY (3α,12α)PREGNAN-20-ONE |
| Q6 | POS | M604T548 | ? |  |  |  |
| Q6 | POS | M299T565 | [M+H]+ |  | C21H30O | 3-Methyl-19-nor-17alpha-pregna-1,3,5(10)-trien-17-ol |
| Q6 | POS | M679T544 | [M+H]+ |  | C40H54O7S | Erythroxanthin sulfate |
| Q6 | POS | M701T544 | [M+Na]+ | | C40H54O7S | Erythroxanthin sulfate |
| Q6 | POS | M315T561 | [M+H]+ |  | C21H30O2 | progesterone or 20a-Hydroxydydrogesterone |
| Q6 | POS | M336T570 | [M+Na]+ | |  | unknown |
| Q6 | POS | M700T545 | [M+H]+ |  | C36H62NO10P | PS(12:0/18:4(6Z,9Z,12Z,15Z)) |
| Q6 | POS | M701T545 | |  | C36H62NO10P | PS(12:0/18:4(6Z,9Z,12Z,15Z)) |
| Q6 | POS | M301T625 | [M+H]+ |  | C21H32O | Allylestrenol or pregn-4-en-3-one or 5alpha-Pregn-2-en-20-one |
| Q6 | POS | M319T623 | [M+H]+ |  | C21H34O2 | Pregn-4-ene-3a,20a-diol or Allopregnanolone or Epi-allopregnanolone |
| Q6 | POS | M461T611 | [M+H]+ |  | C23H36N6O4 | Trp Lys Lys |
| Q6 | POS | M132T112 | [M+ H]+ |  | C4H9N3O2 | Creatine or 3-Guanidinopropionic acid |
| Q6 | POS | M303T611 | [M+H]+ |  | C21H34O | pregnan-21-al or 3beta-Hydroxypregn-5-ene |
| Q6 | POS | M285T611 | [M+H]+ |  | C18H36O2 | Stearic acid |
| Q6 | POS | M286T611 | |  | C18H36O2 | Stearic acid |
| Q6 | POS | M287T612 | |  | C18H36O2 | Stearic acid |
| Q6 | POS | M513T591 | [M+H]+ |  | C27H44O9 | (25S)-11alpha,20,26-trihydroxyecdysone |
| Q6 | POS | M477T625 | [M+H]+ |  | C29H36N2O4 | Ipecac (Emetamine) |
| Q6 | POS | M286T174 | [M+H]+ |  | C17H11N5 | Letrozole |
| Q6 | POS | M497T/611 | [M+H]+ |  | C27H44O8 | Pregnanediol-3-glucuronide |
| Q6 | POS | M514T/611 | [M+NH4]+ | | C27H44O8 | Pregnanediol-3-glucuronide |
| Q6 | POS | M515T611 | |  | C27H44O8 | Pregnanediol-3-glucuronide |
| Q6 | POS | M517T624 | [M+H]+ |  | C25H43NO10 | Mycalamide B |
| Q6 | POS | M519T611 | [M+H]+ |  | C30H38N4O4 | Sativanine B |
| Q6 | POS | M520T611 | |  | C30H38N4O4 | Sativanine B |
| Q6 | POS | M333T561 | [M+H]+ |  | C21H32O3 | Hydroxypregnenolone or ANDROSTERONE ACETATE |
| Q6 | POS | M301T589 | [M+H]+ |  | C18H36O3 | hydroxy-octadecanoic acid |
| Q6 | POS | M519T611 | [M+H]+ |  | C30H46O7 | Cucurbitacin |
| Q6 | POS | M520T611 | |  | C30H46O7 | Cucurbitacin |
| Q6 | POS | M557T611 |  | |  |  |
| Q6 | POS | M558T611 | |  |  |  |
| Q6 | POS | M285T611 | fragment 519.293? | |  |  |
| Q6 | POS | M286T611 | fragment 519.293? | |  |  |
| Q6 | POS | M509T560 | [M+H]+ |  | C27H38F6O2 | hexafluoro-1αa-hydroxyvitamin D3 |
| Q6 | POS | M104T104 | [M+H]+ |  | C5H13NO | Choline or 2-Amino-3-methyl-1-butanol or Neurine |
| Q6 | POS | M363T528 | [M+H]+ |  | C21H30O5 |  |
| Q6 | POS | M317T576 | [M+H]+ |  | C18H36O2S | 2-mercapto-octadecanoic acid |
| Q6 | NEG | M263T118 | [M-H]- |  | C16H8O4 | Frutinone A |
| Q6 | NEG | M494T539 | [M-H]- |  | C25H37NO9 | Dihydrotetrabenazine glucuronide |
| Q6 | NEG | M239T413 | [M-H]- |  | C15H12O3 | Chrysophanic acid 9-anthrone |
| Q6 | NEG | M698T467 | [M-H]- |  | C42H45N5O5 | Adouetine Z |
| Q6 | NEG | M496T534 | [M-H]- |  | C22H44NO9P | PS(16:0/0:0) |
| Q6 | NEG | M632T100 | [M-H]- |  | C34H33FeN4O5 |  |
| Q6 | NEG | M428T105 | ? |  |  |  |
| Q6 | NEG | M493T539 | [M-H]- |  | C29H34O7 | Microlenin or Leukotriene D5 |
| Q6 | NEG | M74T109 | ? |  |  |  |
| Q6 | NEG | M319T107 | [M-H]- |  | C12H24N4O4S1 | Cys Ala Lys |
| Q6 | NEG | M495T535 | [M-H]- |  | C25H40N2O6S | Leukotrien D4 |
| Q6 | NEG | M334T105 | [M-H]- |  | C14H13N3O5S | ISOXICAM |
| Q6 | NEG | M17TT98 | ? |  |  |  |
| Q6 | NEG | M433T104 | [M-H]- |  | C24H18O8 | Sinaticin |
| Q6 | NEG | M363T103 | [M-H]- |  | C13H14Cl2N2O6 | Chloramphenicol 3-acetate |
| Q6 | NEG | M525T444 | [M-H]- |  | C30H38O8 | KHAYASIN C |
| Q6 | NEG | M730T98 | ? |  |  |  |
| Q6 | NEG | M116T116 | [M-H]- |  | C3H7N3O2 | Guanidineacetic acid |
| Q6 | NEG | M523T80 | ? |  |  |  |
| Q6 | NEG | M154T118 | [M-H]- |  | C6H9N3O2 | L-Histidine |
| Q6 | NEG | M155T/108 | [M-H]- |  | C5H4N2O4 | Orotic acid or Uracil 5-carboxylate |
| Q6 | NEG | M309T/113 | [M-H]- |  | C13H14N2O7 |  |
| Q6 | NEG | M881T93 | [M-H]- |  | C49H100NO7P |  |
| Q6 | NEG | M504T109 | ? |  |  |  |
| Q6 | NEG | M509T524 | |  | C30H38O7 | DEOXYGEDUNOL ACETATE |
| Q6 | NEG | M673T102 | ? |  |  |  |
| Q6 | NEG | M506T81 | [M-H]- |  | C10H16N5O13P3 | Adenosine triphosphate (ATP) |
| O3 | POS | M604T548 |  |  | C29H50O10NS |  |
| O3 | POS | M701T570 |  |  | C28H63O4N8P3S | |
| O3 | POS | M700T570 |  |  | C28H63O4N8P3S | |
| O3 | POS | M544T540 | (M+H] |  |  | Cortolone glucuronide |
| O3 | POS | M483T486 | (M+NH4]+ |  | C24H36O9N | estriol glucuronide |
| O3 | POS | M482T487 | (M+NH4] + |  | C24H36O9N | estriol glucuronide |
| O3 | POS | M105T229 |  |  |  |  |
| O3 | POS | M178T39 | [M+Na]1+ |  | C6H9O2N3Na | L-Histidine |
| O3 | POS | M531T630 |  |  | C31H48O4NS | isotope |
| O3 | POS | M528T612 | [M+NH4]1+ |  | C27H46O9N |  |
| O3 | POS | M283T378 |  |  | C12H27O7 |  |
| O3 | POS | M343T714 |  |  | C19H39O3N2 |  |
| O3 | POS | M535T630 |  |  | C33H43O4S |  |
| O3 | POS | M530T630 |  |  | C31H48O4NS |  |
| O3 | POS | M505T609 | [M+Na] + |  | C25H38O9Na | 11-OH-androsterone glucuronide |
| O3 | POS | M479T477 |  |  | ? |  |
| O3 | POS | M327T399 |  |  | C15H27O4N4 |  |
| O3 | POS | M642T511 |  |  | C32H52O12N |  |
| O3 | POS | M100T115 |  |  | C5H10ON |  |
| O3 | NEG | M532T671 | [M+Cl]- |  | C27H44O8Cl | Pregnanediol Glucuronide |
| O3 | NEG | M493T693 |  |  | C27H41O8 |  |
| O3 | NEG | M497T671 | [M+Cl]- |  | C27H44O8Cl | Pregnanediol Glucuronide isotope |
| O3 | NEG | M533T670 |  |  | C27H44O8Cl | Pregnanediol Glucuronide |
| O3 | NEG | M531T671 | [M+Cl]- |  | C27H44O8Cl | Pregnanediol Glucuronide |
| O3 | NEG | M494T693 |  |  | C27H41O8 | isotope |
| O3 | NEG | M602T550 |  |  | C29H48O10NS |  |
| O3 | NEG | M239T504 |  |  | C12H16O3P |  |
| O3 | NEG | M246T528 |  |  | C7H13O3N5P |  |
| O3 | NEG | M509T611 |  |  | C27H41O9 |  |
| O3 | NEG | M714T524 |  |  | C38H57O6N3PS |  |
| O3 | NEG | M569T602 |  |  | ? |  |
| O3 | NEG | M698T570 |  |  | C36H52O9N5 |  |
| O3 | NEG | M463T488 | [M-H] - |  | C24H31O9 | estriol glucuronide |
| O3 | NEG | M464T488 | [M-H] - |  | C24H31O9 | estriol glucuronide |
| O3 | NEG | M603T550 |  |  | C29H48O10NS | isotopes from M602T550 |
| O3 | NEG | M545T593 |  |  | C16H34O16PS |  |
| O3 | NEG | M602T521 |  |  | C32H44O10N |  |
| O3 | NEG | M577T671 |  |  |  | linked to Pregnanediol glucuronide |
| O3 | NEG | M496T671 | [M-H]- |  | C27H44O8Cl | Pregnanediol glucuronide isotope |
| O3 | NEG | M275T80 |  |  | C9H16O2N4PS |  |
| O3 | NEG | M507T590 |  |  | C27H39O9 |  |
| O3 | NEG | M295T546 | [M+Cl]- |  | C6H13O7ClPS | 5-Methylthioribose 1-phosphate |
| O3 | NEG | M256T43 |  |  | C11H15O2NPS |  |
| O3 | NEG | M557T459 |  |  | C20H41O10N6S |  |
| O3 | NEG | M510T631 |  |  | C26H42O5NP2 |  |
| O3 | NEG | M278T454 |  |  | C8H12O8N3 |  |
| O3 | NEG | M415T563 |  |  | ? |  |
| O3 | NEG | M478T478 |  |  | C16H35O10N2P2 | isotope |
| O3 | NEG | M495T671 | [M-H]- |  | C27H43O8 | Pregnanediol glucuronide |
| O3 | NEG | M453T476 |  |  | C17H25O7N8 |  |
| O3 | NEG | M320T512 |  |  |  |  |
| O3 | NEG | M240T535 |  |  | C14H26O2N |  |
| O3 | NEG | M247T46 |  |  | C12H12O2N2P |  |
| O3 | NEG | M316T148 |  |  | C9H19O9NP |  |
| O3 | NEG | M254T148 |  |  | C8H17O6NP |  |
| O3 | NEG | M640T512 |  |  | C33H46O8N5 |  |
| O3 | NEG | M551T683 |  |  | C28H43O7N2S |  |
| O3 | NEG | M477T478 |  |  | C16H35O10N2P2 |  |
| O3 | NEG | M116T52 |  |  | ? |  |
| O3 | NEG | M553T670 |  |  |  | linked to pregnanediol glucuronide |
| Q4 | POS | M85T55 |  |  |  |  |
| Q4 | POS | M381T56 |  |  |  |  |
| Q4 | POS | M427T64 |  |  |  |  |
| Q4 | POS | M563T65 |  |  |  |  |
| Q4 | POS | M178T69 | [M+Na]+ |  | C6H9N3NaO2 | [Histidine](http://maltese.dbs.aber.ac.uk:8888/hrmet/search/printmet.php?metid=D25607) |
| Q4 | POS | M202T70 |  |  |  |  |
| Q4 | POS | M156T70 | [M+H]+ |  | C6H10N3O2 | [Histidine](http://maltese.dbs.aber.ac.uk:8888/hrmet/search/printmet.php?metid=D25607) |
| Q4 | POS | M152T73 | [M+H-HCOOH]+ |  | C2H7N3O3P | [Phosphoguanidinoacetate](http://maltese.dbs.aber.ac.uk:8888/hrmet/search/printmet.php?metid=D25625) |
| Q4 | POS | M332T84 | [M+Na]+ |  | C11H19NNaO9 | [N-Acetylneuraminic acid](http://maltese.dbs.aber.ac.uk:8888/hrmet/search/printmet.php?metid=D629) |
| Q4 | POS | M310T85 | [M+H]+ |  | C11H20NO9 | [N-Acetylneuraminic acid](http://maltese.dbs.aber.ac.uk:8888/hrmet/search/printmet.php?metid=D629) |
| Q4 | POS | M274T91 | [M+H-H2O]+ | | C11H16NO7 | [2-Deoxy-2,3-dehydro-N-acetylneuraminic acid](http://maltese.dbs.aber.ac.uk:8888/hrmet/search/printmet.php?metid=D3767) |
| Q4 | POS | M292T92 | [M+H]+ |  | C11H18NO8 | [2-Deoxy-2,3-dehydro-N-acetylneuraminic acid](http://maltese.dbs.aber.ac.uk:8888/hrmet/search/printmet.php?metid=D3767) |
| Q4 | POS | M158T97 | [M+H]+ |  | C6H8NO4 | [2-Aminomuconic acid](http://maltese.dbs.aber.ac.uk:8888/hrmet/search/printmet.php?metid=D25224) |
| Q4 | POS | M208T115 | [M+H]+ |  | C9H10N3O3 | [Methyl 5-hydroxy-2-benzimidazole carbamate](http://maltese.dbs.aber.ac.uk:8888/hrmet/search/printmet.php?metid=D19610) |
| Q4 | POS | M286T120 | [M+H]+ |  | C12H20N3O5 | [Glycylprolylhydroxyproline](http://maltese.dbs.aber.ac.uk:8888/hrmet/search/printmet.php?metid=D25702) |
| Q4 | POS | M192T289 | [M+H]+ |  | C10H10NO3 | [5-Hydroxyindoleacetic acid](http://maltese.dbs.aber.ac.uk:8888/hrmet/search/printmet.php?metid=D27639) |
| Q4 | POS | M220T307 | [M+H]+ |  | C9H18NO5 | [Pantothenic acid](http://maltese.dbs.aber.ac.uk:8888/hrmet/search/printmet.php?metid=D1169) |
| Q4 | POS | M457T319 |  |  |  |  |
| Q4 | POS | M202T387 | [M+Na]+ |  | C9H9NNaO3 | [Hippuric acid](http://maltese.dbs.aber.ac.uk:8888/hrmet/search/printmet.php?metid=D27604) |
| Q4 | POS | M285T469 | [M+NH4]+ |  | C9H21N2O8 | [Neuraminic acid](http://maltese.dbs.aber.ac.uk:8888/hrmet/search/printmet.php?metid=D26092) |
| Q4 | POS | M170T484 | [M+Na]+ |  | C_9_H_9_NNaO | [3-Methyloxindole](http://maltese.dbs.aber.ac.uk:8888/hrmet/search/printmet.php?metid=D4800) |
| Q4 | POS | M365T547 | [M+H]+ |  | C21H33O5 | [Trihydroxypreg-nenolone](http://maltese.dbs.aber.ac.uk:8888/hrmet/search/printmet.php?metid=D4511) |
| Q4 | POS | M509T571 |  |  |  |  |
| Q4 | POS | M335T656 | [M+H]+ |  | C21H35O3 | [Dihydroxy-5beta-pregnan-20-one](http://maltese.dbs.aber.ac.uk:8888/hrmet/search/printmet.php?metid=D1012) |
| Q4 | POS | M304T676 | [M+NH4]+ |  | C17H38NO3 | [??hydroxy-heptadecanoic acid](http://maltese.dbs.aber.ac.uk:8888/hrmet/search/printmet.php?metid=D10916) |
| Q4 | POS | M305T676 | [M+NH4]^+^ (^13^C) | | C17^13^CH38NO3 | [??hydroxy-heptadecanoic acid](http://maltese.dbs.aber.ac.uk:8888/hrmet/search/printmet.php?metid=D10916) |
| Q4 | POS | M306T676 | [M+NH4]^+^ 2(^13^C) | | C17^13^C2H38NO3 | [??hydroxy-heptadecanoic acid](http://maltese.dbs.aber.ac.uk:8888/hrmet/search/printmet.php?metid=D10916) |
| Q4 | POS | M286T677 | [(M+NH4)-H2O]+ | | C17H36NO2 | [??hydroxy-heptadecanoic acid](http://maltese.dbs.aber.ac.uk:8888/hrmet/search/printmet.php?metid=D10916) |
| Q4 | NEG | M211T56 |  |  |  |  |
| Q4 | NEG | M154T72 | [M-H]- |  | C6H8N3O2 | [Histidine](http://maltese.dbs.aber.ac.uk:8888/hrmet/search/printmet.php?metid=D25607) |
| Q4 | NEG | M435T79 |  |  |  |  |
| Q4 | NEG | M413T79 |  |  |  |  |
| Q4 | NEG | M632T96 |  |  |  |  |
| Q4 | NEG | M319T285 | [M+Cl]- |  | C12H17ClN2O4P | [??-Psilocybin](http://maltese.dbs.aber.ac.uk:8888/hrmet/search/printmet.php?metid=D18789) |
| Q4 | NEG | M218T313 | [M-H]- |  | C9H16NO5 | [Pantothenic acid](http://maltese.dbs.aber.ac.uk:8888/hrmet/search/printmet.php?metid=D1169) |
| Q4 | NEG | M279T320 | [M-H]- |  | C13H15N2O5 | [L-Aspartyl-L-phenylalanine](http://maltese.dbs.aber.ac.uk:8888/hrmet/search/printmet.php?metid=D25986) |
| Q4 | NEG | M274T346 | [M+K-2H]- |  | C7H13KN3O2S2 | [??Cartap](http://maltese.dbs.aber.ac.uk:8888/hrmet/search/printmet.php?metid=D18736) |
| Q4 | NEG | M247T373 | [M+K-2H]- |  | C6H8KO8 | [Glucaric acid](http://maltese.dbs.aber.ac.uk:8888/hrmet/search/printmet.php?metid=D29674) |
| Q4 | NEG | M477T465 |  |  |  |  |
| Q4 | NEG | M463T491 | [M-H]- |  | C24H31O9 | [Estriol-3-glucuronide](http://maltese.dbs.aber.ac.uk:8888/hrmet/search/printmet.php?metid=D23932) |
| Q4 | NEG | M509T547 | [M+Cl]- |  | C32H42ClO3 | [tetranorvitamin D3](http://maltese.dbs.aber.ac.uk:8888/hrmet/search/printmet.php?metid=D22318) |
| Q4 | NEG | M509T621 | [M+Cl]- |  | C32H42ClO3 | [tetranorvitamin D3](http://maltese.dbs.aber.ac.uk:8888/hrmet/search/printmet.php?metid=D22318) |
| Q4 | NEG | M602T613 |  |  |  |  |
| Q4 | NEG | M509T662 | [M+Cl]- |  | C32H42ClO3 | [tetranorvitamin D3](http://maltese.dbs.aber.ac.uk:8888/hrmet/search/printmet.php?metid=D22318) |
| Q4 | NEG | M391T633 | [M+Cl]- |  | C23H32ClO3 | [Acetyloxy-pregna-5,16-dien-20-one](http://maltese.dbs.aber.ac.uk:8888/hrmet/search/printmet.php?metid=D905) |
| Q4 | NEG | M509T603 | [M+Cl]- |  | C32H42ClO3 | [tetranorvitamin D3](http://maltese.dbs.aber.ac.uk:8888/hrmet/search/printmet.php?metid=D22318) |
| Q4 | NEG | M495T683 | [M-H]- |  | C27H43O8 | [Pregnanediol-3-glucuronide](http://maltese.dbs.aber.ac.uk:8888/hrmet/search/printmet.php?metid=D23338) |
| Q4 | NEG | M496T683 | [M-H]^-^ (^13^C) |  | C2713CH43O8 | [Pregnanediol-3-glucuronide](http://maltese.dbs.aber.ac.uk:8888/hrmet/search/printmet.php?metid=D23338) |
| Q4 | NEG | M493T699 |  |  |  | [unknown](http://maltese.dbs.aber.ac.uk:8888/hrmet/search/printmet.php?metid=D21635) |
| Q2 | pos | M203T604 | [M+H]1+ | C15H23 |  | fragment |
| Q2 | pos | M520T604 | [M+Na]1+ | C27H44NaO8 | C27H44O8 | Pregnanediol-3-glucuronide |
| Q2 | pos | M303T604 | [M+H-H20]1+ | C21H35O | C21H36O2 | Pregnanediol (aglycone) |
| Q2 | pos | M233T604 | [M-H +Na2]1+ | C14H3Na2O |  | fragment |
| Q2 | pos | M335T544 | [M+Na]1+ | C25H23O2 |  |  |
| Q2 | pos | M204T586 | [M+H-H2O]1+ | C8H14NO5 | C8H15NO6 | N-Acetyl-D-glucosamine fragment |
| Q2 | pos | M533T508 | [M+Na]1+ | C27H42NaO9 | C21H32O3 | 17alpha-Hydroxypregnenolone (glucuronide) |
| Q2 | pos | M209T604 | [M+NH4]1+ | C3H5N4O5S |  | fragment |
| Q2 | pos | M519T604 | [M+Na]1+ | C27H44NaO8 | C27H44O8 | Pregnanediol-3-glucuronide |
| Q2 | pos | M317T508 | [M+H]1+ | C21H33O2 | C21H32O2 | pregnenolone (aglycone) |
| Q2 | pos | M299T500 | [M+H]1+ | C21H31O | C21H30O3 | 3alpha,21-Dihydroxy-5beta-pregnane-11,20-dione (aglycone) |
| Q2 | pos | M581T605 | [M+CH3OH+H]1+ | C29H41O12 | [C28H36O11](http://www.lipidmaps.org/tools/ms/iso2d_Ag.php?formula=C28H36O11) | Dihydroxy-dimethoxy-prenylflavan-O-β-D-glucopyranoside |
| Q2 | pos | M299T528 | [M+H-H2O]1+ | C21H31O | C21H32O2 | 20α-dihydroprogesterone (aglycone and sulfate) |
| Q2 | pos | M281T528 | [M+H-2H2O]1+ | C21H29 | C21H32O2 | 20α-dihydroprogesterone (aglycone and sulfate) |
| Q2 | pos | M222T492 | [M+H]1+ | C8H16NO6 | C8H15NO6 | N-Acetylhexosamine (fragment) |
| Q2 | pos | M533T528 | [M+Na]1+ | C27H42NaO9 | [C21H34O3](http://www.lipidmaps.org/tools/ms/iso2d_Ag.php?formula=C21H34O3) | 21-hydroxyallopregnanolone glucuronide |
| Q2 | pos | M335T528 | [M+H]1+ | C21H35O3 | [C21H34O3](http://www.lipidmaps.org/tools/ms/iso2d_Ag.php?formula=C21H34O3) | 21-hydroxyallopregnanolone (aglycone) |
| Q2 | pos | M317T529 | [M+H-H2O]1+ | C21H33O2 | [C21H34O3](http://www.lipidmaps.org/tools/ms/iso2d_Ag.php?formula=C21H34O3) | 21-hydroxyallopregnanolone (aglycone) |
| Q2 | pos | M301T568 | [M+H-H2O]1+ | C21H33O | C21H34O2 | 3alpha-Hydroxy-5beta-pregnane-20-on (aglycone) |
| Q2 | pos | M528T541 | [M+H]1+ | C20H34N17O |  |  |
| Q2 | pos | M285T604 | [M+H-H2O]1+ | C21H33 | C21H34O | Pregnan-21-al (aglycone) |
| Q2 | pos | M200T32 | [M+2Na-H]1+ | C6H8N3Na2O2 | C6H9N3O2 | L-histidine |
| Q2 | pos | M204T492 | [M+H-H2O]1+ | C8H16NO6 | C8H15NO6 | N-Acetylhexosamine (fragment) |
| Q2 | pos | M534T546 | [M+H]1+ | C25H44NO9S |  |  |
| Q2 | pos | M283T271 | [M+H]1+ | C12H27O7 |  |  |
| Q2 | pos | M349T292 | [M+Na]1+ | C14H30NaO8 |  |  |
| Q2 | pos | M656T38 | [M+2Na-H]1+ | C20H24N19Na2O5 |  |  |
| Q2 | pos | M327T292 | [M+H]1+ | C14H31O8 |  |  |
| Q2 | pos | M165T392 | [M+H]1+ | C10H13O2 | C_10_H_12_O_2_ | Isoeugenol |
| Q2 | pos | M533T546 | [M+K]1+ | C21H38KN10O4 |  |  |
| Q2 | pos | M305T271 | [M+Na]1+ | C12H26NaO7 |  |  |
| Q2 | pos | M299T546 | [M+H-2H2O]1+ | C21H31O | C21H34O3 | 3alpha,12alpha-Dihydroxy-5beta-pregnan-20-one (aglycone) |
| Q2 | pos | M110T25 | [M+H-HCO2]1+ | C5H8N3 | C6H9N3O2 | decarboxylated L-Histidine |
| Q2 | pos | M279T392 | [M+H]1+ | C16H23O4 | C16H22O4 | Diisobutyl phthalate |
| Q2 | neg | M561T635 | [M+CF3COOH-H]1- | C20H38F5N4O4 |  |  |
| Q2 | neg | M510T529 | [M-H]1- | C27H41O9 |  |  |
| Q2 | neg | M604T587 | [M-H]1- | C18H42N11O12 |  |  |
| Q2 | neg | M577T529 | [M-H]1- | C25H41N2O13 |  |  |
| Q2 | neg | M603T554 | [M+Na-2H]1- | C24H45N5NaO11 |  |  |
| Q2 | neg | M415T680 | [M+Na-2H]1- | C16H32N4NaO7 |  |  |
| Q2 | neg | M525T435 | [M-H]1- | C23H37N6O8 |  |  |
| Q2 | neg | M603T586 | [M-H]1- | C26H51O13S |  |  |
| Q2 | neg | M413T741 | [M-H]1- | C5HO20S |  |  |
| Q2 | neg | M509T529 | [M-H]1- | C27H41O9 | [C27H42O9](http://www.chemspider.com/Molecular-Formula/C27H42O9) | 17-Hydroxy-20-oxopregnan-3-yl β-D-glucopyranosiduronic acid |
| Q2 | neg | M365T561 | [M-H]1- | C18H21O6S |  | unknown |
| Q2 | neg | M523T435 | [M+CH3COOH-H]1- | C27H39O10 | C25H36O8 | Dehydroisoandrosterone 3-glucuronide |
| Q2 | neg | M602T585 | [M-H]1- | C29H48NO10S |  | unknown |
| Q2 | neg | M201T156 | [M-H]1- | C9H13O5 | [C8H12O3](http://www.lipidmaps.org/tools/ms/iso2d_Ag.php?formula=C8H12O3) | 5-oxo-7-octenoic acid |
| Q2 | neg | M493T627 | [M-H]1- | C27H41O8 | [C27H42O8](http://www.chemspider.com/Molecular-Formula/C27H42O8) | 14-Hydroxy-20-oxopregn-5-en-3-yl β-D-glucopyranoside |
| Q2 | neg | M508T503 | isotope |  |  | unknown |
| Q2 | neg | M693T604 | [M-H2O-H]1- | C19H33N16O13 |  | unknown |
| Q2 | neg | M399T506 | [M-H]1- | C23H19N4O3 |  | unknown |
| Q2 | neg | M349T778 | [M-H]1- | C18H21O5S | C18H22O5S | Estrone sulfate |
| Q2 | neg | M602T553 | [M-H]1- | C25H41N13OPS |  |  |
| Q2 | neg | M699T604 | [M-H]1- | C27H43O8+(CF3)3 | C_27_H_44_O_8_ | Pregnanediol-3-glucuronide |
| Q2 | neg | M698T492 | [M+Na-2H]1- | C33H46FN13NaO2 |  |  |
| Q2 | neg | M557T444 | [M+CF3COOH-H]1- | C21H23F10N2O2S |  |  |
| Q2 | neg | M449T785 | [M+Na-2H]1- | C18H18NaO10S |  |  |
| Q2 | neg | M509T506 | [M+Na-2H]1- | C18H39N12NaPS |  |  |
| Q2 | neg | M481T663 | [M-H]1- | C21H33O3+2CF3-2H | C21H34O3 | 3alpha,17alpha-Dihydroxy-5beta-pregnan-20-one |
| Q2 | neg | M511T537 | [M-H]1- | C27H43O9 | [C27H44O9](http://www.chemspider.com/Molecular-Formula/C27H44O9) | 3,21-Dihydroxypregnan-20-yl β-D-glucopyranosiduronic acid |
| Q2 | neg | M690T477 | [M+CF3COOH-H]1- | C27H52F4NO12S |  |  |
| Q2 | neg | M578T547 |  |  | isotope |  |
| Q2 | neg | M437T791 | [M+CF3COOH-H]1- | C15H13F4N4O5S |  |  |
| Q2 | neg | M381T554 | [M+CF3COOH-H]1- | C14H17F4N4O2S |  |  |
| Q2 | neg | M414T663 |  |  | isotope |  |
| Q2 | neg | M523T453 | [M-H]1- | C27H39O10 | [C27H40O10](http://www.chemspider.com/Molecular-Formula/C27H40O10) | 11,17-Dihydroxy-3,20-dioxopregn-4-en-21-yl hexopyranoside |
| O1 | POS | M132T54 |  |  |  | Creatine |
| O1 | POS | M150T49 |  |  |  |  |
| O1 | POS | M199T289 |  |  |  |  |
| O1 | POS | M204T422 |  |  |  | N2-Acetyl-L-aminoadipate |
| O1 | POS | M209T291 |  |  |  |  |
| O1 | POS | M219T49 |  |  |  | cis-1,2-Dihydroxy-1,2-dihydrodibenzothiophene |
| O1 | POS | M245T54 |  |  |  |  |
| O1 | POS | M270T298 |  |  |  | Nefopam N-oxide ore or |
|  |  |  |  |  |  | Methyl-(tetrahydro-methoxy-naphthyl)-2(1H)-pyridon or |
|  |  |  |  |  |  | Dimethoxy-[2-(4-Pyridinyl)-1-butenyl]phenol |
| O1 | POS | M279T371 |  |  |  | 2-Ethylhexylphthalate or Dibutylphthalate or Diisobutylphthalat |
| O1 | POS | M280T371 |  |  |  | Ser Ser Ser |
| O1 | POS | M280T371 |  |  |  | Oxamniquine |
| O1 | POS | M287T507 |  |  |  |  |
| O1 | POS | M300T303 |  |  |  | Ethylketocyclazocine |
| O1 | POS | M317T432 |  |  |  | Pregnenolone or 5alpha-Pregnane-3,20-dione or |
|  |  |  |  |  |  | Hydroxypregn-one or alpha-Hydroxy-pregnen-one |
| O1 | POS | M332T53 |  |  |  |  |
| O1 | POS | M335T448 |  |  |  | 21-hydroxyallopregnanolone or Tetrahydrodeoxycorticosterone |
|  |  |  |  |  |  | or 3alpha,6alpha-Dihydroxy-5beta-pregnan-20-one |
| O1 | POS | M336T448 |  |  |  |  |
| O1 | POS | M363T403 |  |  |  | 9alpha-Fluoro-11beta-hydroxy-6alpha-methylpregn-4-ene-3,20-dione |
|  |  |  |  |  |  | or 4-Fluoro-17beta-hydroxyandrost-4-en-3-onepropionate or |
|  |  |  |  |  |  | 16beta-Fluoro-17beta-hydroxyandrost-4-en-3-onepropionate |
| O1 | POS | M418T309 |  |  |  | decoquinate |
| O1 | POS | M462T316 |  |  |  |  |
| O1 | POS | M462T430 |  |  |  |  |
| O1 | POS | M495T514 |  |  |  | Thiobinupharidine |
| O1 | POS | M509T433 |  |  |  | 26,26,26,27,27,27-hexafluoro-1alphaa-hydroxyvitamin D3 |
| O1 | POS | M511T456 |  |  |  |  |
| O1 | POS | M512T456 |  |  |  | 4-Hydroxymibefradil |
| O1 | POS | M513T471 |  |  |  |  |
| O1 | POS | M528T456 |  |  |  |  |
| O1 | POS | M539T383 |  |  |  |  |
| O1 | POS | M604T414 |  |  |  |  |
| O1 | POS | M605T414 |  |  |  |  |
| O1 | POS | M629T282 |  |  |  | Resiniferatoxin |
| O1 | POS | M642T385 |  |  |  | b-D-Glucopyranosiduronic acid, (3a,5b,7a,12a)-24- |
|  |  |  |  |  |  | 24-[(carboxymethyl)amino]-1,12-dihydroxy-24-oxocholan-3-yl |
| O1 | POS | M643T385 |  |  |  | glycoprotein1,2-didecanoyl-sn-phosphatidylinositol |
| O1 | POS | M690T497 |  |  |  |  |
| O1 | POS | M690T497 |  |  |  |  |
| O1 | POS | M691T497 |  |  |  |  |
| O1 | POS | M700T422 |  |  |  | LasiodineA |
| O1 | POS | M700T422 |  |  |  |  |
| O1 | POS | M700T422 |  |  |  |  |
| O1 | POS | M701T422 |  |  |  | Chalcomycin |
| O1 | POS | M701T422 |  |  |  |  |
| O1 | POS | M701T422 |  |  |  |  |
| O1 | POS | M702T422 |  |  |  | LeucomycinV |
| O1 | POS | M716T390 |  |  |  |  |
| O1 | POS | M716T390 |  |  |  |  |
| O1 | POS | M88T248 |  |  |  | 3-Methylbutanamine |
| O1 | POS | M98T155 |  |  |  |  |
| Q3 | POS | M215T0 | [M+K]+ 175.956 [M+Na]+ 191.932 | |  |  |
| Q3 | POS | M233T0 |  |  |  |  |
| Q3 | POS | M85T0 | [M+2Na+2K-H]3+ 131.99 [M+2Na+2K-H]3+ 131.99 | | |  |
| Q3 | POS | M103T0 |  |  |  |  |
| Q3 | POS | M187T0 |  |  |  |  |
| Q3 | POS | M251T0 |  |  |  |  |
| Q3 | POS | M152T1 | [M+K]+ |  |  | Creatinine (and isomer) |
| Q3 | POS | M165T1 |  |  |  |  |
| Q3 | POS | M166T1 | [(M+H)-(C5H8O4)]+ | |  | N2-methylguanosine |
| Q3 | POS | M220T5 | [M+H]1+ |  |  | Pantothenic acid |
| Q3 | POS | M275T6 |  |  |  |  |
| Q3 | POS | M319T7 |  |  |  |  |
| Q3 | POS | M308T10 |  |  |  |  |
| Q3 | POS | M267T11 | [M+H]+ |  |  | Phenylacetylglutamine |
| Q3 | POS | M509T11 |  |  |  |  |
| Q3 | POS | M285T11 |  |  |  |  |
| Q3 | POS | M170T11 |  |  |  |  |
| Q3 | POS | M271T12 |  |  |  |  |
| Q3 | POS | M447T12 |  |  |  |  |
| Q3 | POS | M253T12 |  |  |  |  |
| Q3 | POS | M563T12 |  |  |  |  |
| Q3 | POS | M317T12 | [M+H]+ |  |  | Decanoylcarnitine isomer |
| Q3 | POS | M317T12 | [M+H]+ |  |  | Decanoylcarnitine (13C) |
| Q3 | POS | M316T12 | [M+H]+ |  |  | Decanoylcarnitine isomer |
| Q3 | POS | M316T12 | [M+H]+ |  |  | Decanoylcarnitine |
| Q3 | POS | M333T12 |  |  |  |  |
| Q3 | POS | M204T12 | [M+H]+ |  |  | (ethoxycarbonyl)-hydroxypyrrolidine-carboxylic acid_ |
|  |  |  |  |  |  | (methoxyacetyl)-hydroxypyrrolidine-carboxylic acid |
| Q3 | POS | M204T12 | [M+H]+ |  |  | N2-acetylaminoadipic acid |
| Q3 | POS | M204T12 | [M+H]+ |  |  | ethoxycarbonyl)-hydroxypyrrolidine-carboxylic acid_ |
|  |  |  |  |  |  | (methoxyacetyl)-hydroxypyrrolidine-carboxylic acid |
| Q3 | POS | M204T13 |  |  |  |  |
| Q3 | POS | M281T13 |  | |  |  |
| Q3 | POS | M301T13 |  |  |  |  |
| Q3 | POS | M299T13 |  |  |  |  |
| Q3 | POS | M285T14 |  |  |  |  |
| O4 | POS | M534T9 |  |  |  |  |
| O4 | POS | M299T9 |  |  |  | 3-Methyl-19-nor-17alpha-pregna-1,3,5(10)-trien-17-ol |
| O4 | POS | M285T9 |  |  |  |  |
| O4 | POS | M204T8 |  |  |  | Tritophanamide or Aminoantipyrine |
| O4 | POS | M317T9 |  |  |  | Pregnenolone; Calusterone; Bolasterone; 5α-dihydroprogesterone; |
|  |  |  |  |  |  | (20S)-20-hydroxypregn-4-en-3-one;  5beta-pregnane-3,20-dione; Ethyltestosterone; |
|  |  |  |  |  |  | 17beta-Hydroxy-2,17-dimethyl-5alpha-androst-1-en-3-one; |
|  |  |  |  |  |  | Calusterone; Norbolethone; Urushiol III;  17-Hydroxy-5alpha,17alpha-pregn-1-en-3-one; |
|  |  |  |  |  |  | 17beta-Hydroxy-6alpha,17-dimethylandrost-4-en-3-one; |
|  |  |  |  |  |  | 3-Acetyl-5alpha-androst-2-en-17beta-ol;  3-Ethynyl-5alpha-androstane-3beta,17beta-diol; |
|  |  |  |  |  |  | 17beta-Hydroxy-4,4-dimethylandrost-5-en-3-one; |
|  |  |  |  |  |  | 17alpha,2alpha-Dimethyl-17beta-hydroxy-4-androsten-3-one |
| O4 | POS | M365T1 |  |  |  | Maltose; Lactulose; Lactose; IsoMaltose; sucrose |
| O4 | POS | M164T1 |  |  |  |  |
| O4 | POS | M541T9 |  |  |  | hexafluoro-seco-cholestatriene-tetrol |
| O4 | POS | M302T9 |  |  |  | pregnan-21-al; (15:1)-Cardanol; 3beta-Hydroxypregn-5-ene |
| O4 | POS | M533T9 |  |  |  | Calotropin; Roridin A |
| O4 | POS | M204T9 |  |  |  | N2-Acetyl-L-aminoadipate **or** n-Hydroxyadipic acid |
| O4 | POS | M281T9 |  |  |  | 3-Methyl-19-nor-17alpha-pregna-1,3,5(10)-trien-17-ol |
| O4 | POS | M381T1 |  |  |  | Veratric acid glucuronide **or** N5-Carboxyaminoimidazole ribonucleotide |
| O4 | POS | M527T8 |  |  |  | 16a-Hydroxydydrogesterone glucuronide; |
|  |  |  |  |  |  | 21-Hydroxydydrogesterone glucuronide; 20-acetoxy-clavulone |
|  |  |  |  |  |  | Allylestrenol; pregn-4-en-3-one; 5alpha-Pregn-2-en-20-one;  17-Ethynyl-5alpha-androstan-17beta-ol |
| O4 | POS | M301T9 |  |  |  |  |
| O4 | POS | M536T9 |  |  |  |  |
| O4 | POS | M93T1 |  |  |  |  |
| O4 | POS | M349T2 |  |  |  | Ser Ser Arg; Arg Ser Ser ; Ser Arg Ser |
| O4 | POS | M334T8 |  |  |  | 17α-hydroxypregnenolone; 5α-dihydrodeoxycorticosterone; |
|  |  |  |  |  |  | (20S)-1α,20-dihydroxy-22,23,24,25,26,27-hexanorvitamin D3; |
|  |  |  |  |  |  | Oxymetholone C21H32O3; ANDROSTERONE ACETATE; |
|  |  |  |  |  |  | Leukotriene A4 methyl ester, Tintinnadiol;  (20R)-17,20-dihydroxypregn-4-en-3-one; |
|  |  |  |  |  |  | 17,20-dihydroxypregn-4-en-3-one; 21-hydroxypregnenolone; |
|  |  |  |  |  |  | (20S)-17,20-dihydroxypregn-4-en-3-one;  3a,7a-Dihydroxy-5b-cholestanate; |
|  |  |  |  |  |  | 5(6)-EpETE methyl ester; 7α-Hydroxypregnenolone; |
|  |  |  |  |  |  | 5α-Dihydrodeoxycorticosterone; Alphaxalone; |
|  |  |  |  |  |  | 11beta,17beta-Dihydroxy-6alpha,17-dimethylandrost-4-en-3-one; |
|  |  |  |  |  |  | 11alpha,17beta-Dihydroxy-2alpha,17-dimethylandrost-4-en-3-one; |
|  |  |  |  |  |  | 11alpha-Hydroxy-5beta-pregnane-3,20-dione; |
|  |  |  |  |  |  | 12alpha-Hydroxy-5beta-pregnane-3,20-dione; |
|  |  |  |  |  |  | 11beta,17beta-Dihydroxy-4,17-dimethylandrost-4-en-3-one; |
|  |  |  |  |  |  | 17beta-Carbomethoxyandrost-5-en-3beta-ol; |
|  |  |  |  |  |  | 3beta,6alpha-dihydroxy-5alpha-pregn-9(11)-en-20-one; |
|  |  |  |  |  |  | 16-a-Hydroxypregnenolone; 17a-Hydroxypregnolone; |
|  |  |  |  |  |  | 21-Hydroxypregnenolone |
| O4 | POS | M535T9 |  |  |  | Corotoxigenin-3-O-alpha-L-rhamnopyranoside; Helveticoside |
| O4 | POS | M335T9 |  |  |  | 21-hydroxyallopregnanolone; dihydroxy(3α,12α)pregnan-20-one; |
|  |  |  |  |  |  | Leukotriene A3 methyl ester; Tetrahydrodeoxycorticosterone; |
|  |  |  |  |  |  | (±)5(6)-EET methyl ester;(±)8(9)-EET methyl ester;  (±)11(12)-EET methyl ester; |
|  |  |  |  |  |  | (±)14(15)-EET methyl ester;  3alpha,6alpha-Dihydroxy-5beta-pregnan-20-one; |
|  |  |  |  |  |  | 3alpha,17alpha-Dihydroxy-5beta-pregnan-20-one; |
|  |  |  |  |  |  | 17beta-Hydroxy-2alpha-(hydroxymethyl)-17-methyl-5alpha  -androstan-3-one; |
|  |  |  |  |  |  | D-Homo-17a-oxa-5alpha-androstan-3beta-ol acetate; |
|  |  |  |  |  |  | Asterogenol; Tetrahydrodeoxycorticosterone |
| O4 | POS | M292T1 |  |  |  | ? |
| O4 | POS | M379T8 |  |  |  | 6β-Hydroxycortisol; 18-Hydroxycortisol; |
|  |  |  |  |  |  | 11,17,18,21-tetrahydroxy-pregn-4-ene-3,20-dione |
| O4 | POS | M363T7 |  |  |  | 18-Hydroxycorticosterone; 4,5β-Dihydrocortisone; 4,5α-Dihydrocortisone; |
|  |  |  |  |  |  | 1α,17α,21-trihydroxy-20-oxo-22,23,24,25,26,27-hexanorvitamin D3; |
|  |  |  |  |  |  | Humulone; 16alpha-Hydroxycorticosterone; Cortisol; |
|  |  |  |  |  |  | 11b,21-Dihydroxy-3,20-oxo-5b-pregnan-18-al; |
|  |  |  |  |  |  | 20β-Dihydroprednisolone; 20α-Dihydroprednisolone |
| O4 | POS | M284T9 |  |  |  |  |
| O4 | POS | M191T1 |  |  |  |  |
| O4 | POS | M178T1 |  |  |  |  |
| O4 | POS | M157T1 |  |  |  |  |
| O4 | POS | M189T10 |  |  |  |  |
| O4 | POS | M354T1 |  |  |  | Glu Cys Cys; Cys Glu Cys; Cys Cys Glu |
| O4 | POS | M459T9 |  |  |  | Prednisolone tebutate; Lucidenic acid A |
| O4 | POS | M315T6 |  |  |  | Progesterone; 3alpha-Ethynyl-3-hydroxy-5alpha-androstan-17-one; |
|  |  |  |  |  |  | 5beta-Pregn-11-ene-3,20-dione; |
|  |  |  |  |  |  | 17beta-Hydroxy-2alpha,17-dimethyl-4,9(11)-androstadien-3-one; |
|  |  |  |  |  |  | 3,5-Cyclo-5alpha,17alpha-pregn-20-yne-6beta,17-diol; |
| O4 | POS | M276T1 |  |  |  |  |
| O4 | POS | M194T1 |  |  |  |  |
| O4 | POS | M205T7 |  |  |  |  |
| O4 | POS | M475T9 |  |  |  | Diterpenoid EF-D; GITOXIGENIN DIACETATE; Lucidenic acid |
| O4 | POS | M642T7 |  |  |  | [(carboxymethyl)amino]-dihydroxy-oxocholan-yl-b-  D-Glucopyranosiduronic acid |
| O4 | POS | M180T1 |  |  |  |  |
| O4 | POS | M222T7 |  |  |  |  |
| O4 | POS | M557T9 |  |  |  |  |
| O4 | POS | M479T7 |  |  |  | Okanin 3,4-dimethyl ehter 4'-glucoside; |
|  |  |  |  |  |  | 2',4',4-Trihydroxy-3',3-dimethoxychalcone 4'-O-glucoside; |
|  |  |  |  |  |  | Phloretin 2'-O-(2''-O-Acetylglucoside); |
|  |  |  |  |  |  | Eriodictyol 3',4'-dimethyl ether 5-O-glucoside; |
|  |  |  |  |  |  | 7,3',4'-Trihydroxy-5-methoxy-6-C-methylflavanone 7-O-glucoside; |
|  |  |  |  |  |  | Eriodictyol 5,4'-dimethyl ether 7-O-glucoside; |
|  |  |  |  |  |  | Persiconin; Lindleyin; Persicogenin 3'-glucoside; |
|  |  |  |  |  |  | 4'-O-methyl-(-)-epicatechin-5-O-beta-glucuronide; |
| O4 | POS | M186T7 |  |  |  |  |
| O4 | POS | M369T8 |  |  |  | 3-β-hydroxyandrost-5-en-17-one sulfate; DHEA sulfate; |
|  |  |  |  |  |  | Testosterone sulfate; prasterone sulfate |
| N1 |  | 1.495 |  |  |  | Alanine |
| N1 |  | 1.335 |  |  |  | Lactate ou threonine |
| N1 |  | 1.485 |  |  |  | Alanine |
| N1 |  | 4.455 |  |  |  | carnosine |
| N1 |  | 1.345 |  |  |  | Lactate ou threonine |
| N1 |  | 1.115 |  |  |  | Isobutyrate |
| N1 |  | 1.105 |  |  |  | Isobutyrate |
| N1 |  | 4.5 |  |  |  |  |
| N1 |  | 7.905 |  |  |  | carnosine |
| N1 |  | 7.105 |  |  |  | carnosine |
| N1 |  | 4.15 |  |  |  | proline |
| N1 |  | 4.465 |  |  |  | carnosine |
| N1 |  | 1.475 |  |  |  | Alanine |
| N1 |  | 4.475 |  |  |  | carnosine |
| N1 |  | 1.95 |  |  |  | Lysine |
| N1 |  | 4.265 |  |  |  | threonine |
| N1 |  | 1.325 |  |  |  | Lactate or threonine |
| N1 |  | 3.855 |  |  |  |  |
| N1 |  | 3.565 |  |  |  | Glycine |
| N1 |  | 2.455 |  |  |  | Glutamine |
| N1 |  | 3.795 |  |  |  |  |
| N1 |  | 8.895 |  |  |  | N-methylnicotinic acid |
| N1 |  | 3.805 |  |  |  |  |
| N1 |  | 1.905 |  |  |  | Lysine |
| N1 |  | 0.805 |  |  |  |  |
| N1 |  | 2.245 |  |  |  | aminoadipic acid |
| N1 |  | 8.905 |  |  |  | N-methylnicotinic acid |
| N1 |  | 0.635 |  |  |  |  |
| N1 |  | 2.945 |  |  |  | asparagine |
| N1 |  | 3.995 |  |  |  | asparagine |
| N1 |  | 3.35 |  |  |  |  |
| N1 |  | 0.665 |  |  |  |  |
| N1 |  | 1.405 |  |  |  |  |
| N1 |  | 8.95 |  |  |  | N-methylnicotinic acid |
| N1 |  | 0.785 |  |  |  |  |
| N1 |  | 0.925 |  |  |  |  |
| N1 |  | 2.155 |  |  |  | Glutamine |
| N1 |  | 1.395 |  |  |  |  |
| N1 |  | 7.915 |  |  |  | carnosine |
| N1 |  | 4.275 |  |  |  | threonine |
| N1 |  | 4.255 |  |  |  | Threonine |
| N2 |  | 1.46 - 1.50 |  |  |  | Alanine |
| N2 |  | 1.30 - 1.36 |  |  |  | Threonine or lactate |
| N2 |  | 1.10 - 1.14 |  |  |  | 2-oxoisovaleric acid |
| N2 |  | 3.54 - 3.58 |  |  |  | Glycine |
| N2 |  | 3.58 - 3.62 |  |  |  | Threonine |
| N2 |  | 3.78 - 3.82 |  |  |  |  |
| N3 |  | 2.58055007 |  |  |  | citrate |
| N3 |  | 0.76550001 |  |  |  |  |
| N3 |  | 2.70614994 |  |  |  | citrate + DMA |
| N3 |  | 8.56669998 |  |  |  |  |
| N3 |  | 3.75985003 |  |  |  | Glutamine |
| N3 |  | 3.12240005 |  |  |  | Creatinine |
| N5 |  | 0.5699 |  |  |  | endogenous cholic acids |
| N5 |  | 1.33775 |  |  |  | endogenous, lactate/threonate |
| N5 |  | 1.4907 |  |  |  | endogenous alanine + low levels signals from spiked cpds |
| N5 |  | 1.10805 |  |  |  | endogenous, methyl succinate (doublet 1.11 ppm) |
| N5 |  | 0.7992 |  |  |  | endogenous cholic acids |
| N5 |  | 4.0041 |  |  |  | low levels signals from spiked cpds |
| N5 |  | 3.5732 |  |  |  | glycine |
| N5 |  | 0.77145 |  |  |  | endogenous cholic acids |
| N5 |  | 0.55775 |  |  |  | endogenous cholic acids |
| N5 |  | 0.5332 |  |  |  | endogenous cholic acids |
| N5 |  | 3.8688 |  |  |  | low levels signals from spiked cpds |
| N5 |  | 2.45485 |  |  |  | pyroglutamate/riboflavin |
| N5 |  | 3.80505 |  |  |  | endogenous, small signals |
| N5 |  | 8.96555 |  |  |  | endogenous, signals |
| N5 |  | 1.9105 |  |  |  | low levels signals from spiked cpds |
| N5 |  | 8.8998 |  |  |  | endogenous, signals |
|  |  |  |  |  |  |  |

**Table 5.** Urine densities and dilution correction factors

| **Sample identifier** | **Specific gravity** | **Normalization factor*** |
| --- | --- | --- |
| 1 | 1.0046 | 3.5067 |
| 2 | 1.0087 | 1.8541 |
| 3 | 1.0214 | 0.7538 |
| 4 | 1.0047 | 3.4321 |
| 5 | 1.0092 | 1.7533 |
| 6 | 1.018 | 0.8962 |
| 7 | 1.0245 | 0.6584 |
| 8 | 1.0219 | 0.7366 |
| 9 | 1.0229 | 0.7044 |
| 10 | 1.0267 | 0.6041 |
| 11 | 1.0232 | 0.6953 |
| 12 | 1.017 | 0.9489 |
| 13 | 1.0069 | 2.3378 |
| Average | 1.0161 |  |

* calculated as (average specific gravity-1)/(sample specific gravity-1).
